# Evaluation of Keratin–Cellulose Blend Fibers as Precursors for Carbon Fibers

**DOI:** 10.1021/acssuschemeng.2c00976

**Published:** 2022-06-22

**Authors:** Hilda Zahra, Julian Selinger, Daisuke Sawada, Yu Ogawa, Hannes Orelma, Yibo Ma, Shogo Kumagai, Toshiaki Yoshioka, Michael Hummel

**Affiliations:** † Department of Bioproducts and Biosystems, Aalto University, Vuorimiehentie 1, 02150 Espoo, Finland; ‡ Institute of Bioproducts and Paper Technology, Graz University of Technology, Inffeldgasse 23, 8010 Graz, Austria; § Univ. Grenoble Alpes, CNRS, CERMAV, 38000 Grenoble, France; ∥ VTT Technical Research Centre of Finland Ltd., Biomaterial Processing and Products, Tietotie 4E, 02044 Espoo, Finland; ⊥ Graduate School of Environmental Studies, Tohoku University, 6-6-07 Aoba, Aramaki-aza, Aoba-ku, 980-8579 Sendai, Japan; # Division for the Establishment of Frontier Sciences of Organization for Advanced Studies, Tohoku University, 2-1-1 Katahira, Aoba-ku, 980-8577 Sendai, Japan

**Keywords:** cellulose, keratin, composite fiber, carbon fiber, synergistic effect, pyrolysis, carbon nanostructure

## Abstract

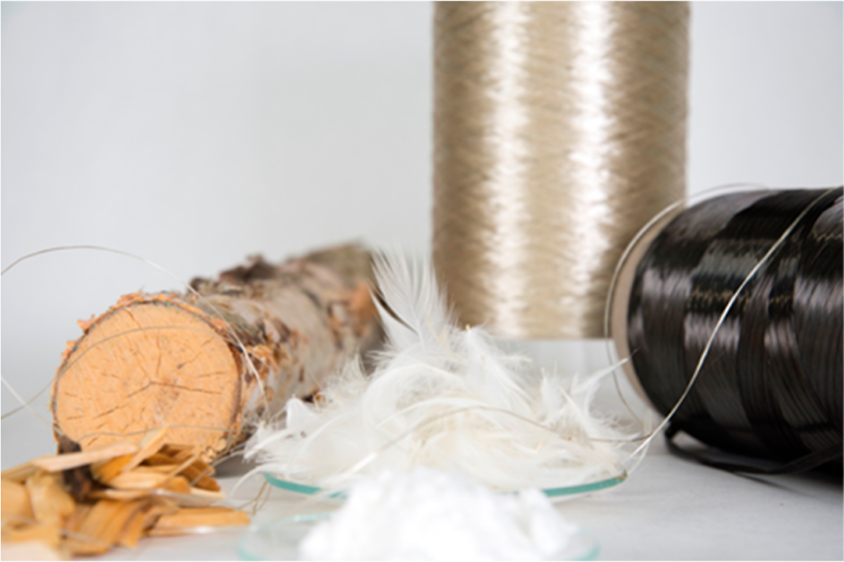

One main challenge to utilize cellulose-based fibers as the precursor for carbon fibers is their inherently low carbon yield. This study aims to evaluate the use of keratin in chicken feathers, a byproduct of the poultry industry generated in large quantities, as a natural charring agent to improve the yield of cellulose-derived carbon fibers. Keratin–cellulose composite fibers are prepared through direct dissolution of the pulp and feather keratin in the ionic liquid 1,5-diazabicyclo[4.3.0]non-5-enium acetate ([DBNH]OAc) and subsequent dry jet wet spinning (so-called Ioncell process). Thermogravimetric analysis reveals that there is an increase in the carbon yield by ∼53 wt % with 30 wt % keratin incorporation. This increase is comparable to the one observed for lignin–cellulose composite fibers, in which lignin acts as a carbon booster due to its higher carbon content. Keratin, however, reduces the mechanical properties of cellulose precursor fibers to a lesser extent than lignin. Keratin introduces nitrogen and induces the formation of pores in the precursor fibers and the resulting carbon fibers. Carbon materials derived from the keratin–cellulose composite fiber show potential for applications where nitrogen doping and pores or voids in the carbon are desirable, for example, for low-cost bio-based carbons for energy harvest or storage.

## Abstract 2

This paper elucidates the role of keratin derived from chicken feathers, a major waste stream of the poultry industry, as a natural catalyst to increase the yield of cellulose-based carbon fibers.

## Introduction

Carbon fibers (CFs) provide superior properties as reinforcement for composites: lightweight, excellent mechanical properties, high stiffness, and resistance toward stress corrosion or failure, low thermal expansion, and good electrical and thermal conductivity.^1,2^ Their application has been mainly developed for specialized sectors such as aerospace, aircrafts, and automotive industries. Its widespread use in large-volume and low-price market segments (e.g., sporting goods, electronic equipment, and constructing materials) is still hampered by the high cost of its main precursor polymers (polyacrylonitrile), which can account for up to 50% of the total CF cost.^3^ Carbonization is an energy-intensive process and stabilization of the precursor fibers at a low heating rate can contribute to 30–40% of total energy cost.^3^ Consequently, the use of low-cost and renewable precursors for CFs has become the subject of intense research to meet the growing demand on the CF market.

Besides their high strength and uniformity,^4−6^ man-made cellulosic fibers (MMCFs) are suitable as CF precursors because they could be produced in a large volume from low-cost and high-purity cellulosic materials.^7^ However, the uneconomically low CF yield (∼10 wt %) after cellulose pyrolysis has been a hindrance for the industrial production of cellulose-based CF. The significantly lower carbon yield from the theoretical maximum (∼44 wt %) is due to the formation of levoglucosan and other carbonaceous volatiles upon thermal depolymerization of cellulose.^8,9^ The common strategy to suppress the formation of levoglucosan is by promoting dehydration reactions during cellulose pyrolysis.^7^ Among several proposed strategies, incorporating bio-based catalysts into the cellulose fibers, could be a simple, practical, and sustainable route to promote the dehydration reaction and consequently increase the carbon yield.

In our previous report, chitosan, an amine-containing cellulose analogue and a natural charring agent, substantially improved the yield of the CFs derived from cellulose–chitosan blend fibers.^10^ This type of mixed polymer fibers, herein called composite fibers, was produced via a newly developed Lyocell-type spinning process termed Ioncell. In this process, cellulose and chitosan were co-dissolved in a non-derivatizing solvent diazabicyclo[4.3.0]non-5-enium acetate ([DBNH]OAc) and then spun and regenerated in aqueous spin bath. The structural similarity and compatibility between chitosan and cellulose result in a homogeneous distribution of chitosan in between cellulose chains, bringing both biopolymers in intimate contact. This enhances the catalytic activity of the amine group in chitosan in slowing down the cellulose degradation and enhancing the carbon yield.^11^

In this study, we evaluate the use of keratin derived from chicken feathers as catalysts^12^ to improve the yield of the cellulose-based CF. From an environmental point of view, it is attractive because chicken feather waste is generated in large amounts by the poultry industry.^13^ Feather keratin is a fibrous protein that is largely stabilized by disulfide bridges,^14^ and its dissolution in organic solvents is challenging.^13^ In this work, keratin–cellulose composite fibers are prepared via the Ioncell process, involving the direct dissolution of both biopolymers in [DBNH]OAc. We first investigate the distribution of keratin in the composite fibers and its effect on the structural, mechanical, and thermochemical properties of the composite fibers. Thereafter, we discuss the effect of keratin and the carbonization temperature on the structural properties and electrical conductivity of the resulting cellulose-based CFs.

## Materials and Methods

### Preparation of Keratin–Cellulose Composite Fibers

Cellulose pulp was obtained from grounded prehydrolysis kraft birch (*Betula pendula*) pulp sheets ([η] = 494 mL/g, *M*_n_ = 72.9 kDa, *M*_w_ = 262.9 kDa, polydispersity 3.6, Stora Enso Enocell, Finland). Chicken feathers (HK-Scan, Eura, Finland) were successively treated with ethanol, water, detergent agent (Dexonex 22HPX-X, 3% consistency), and water. The feathers were then dried, autoclaved, and stored at −20 °C. Prior to use, the feathers were dried at 60 °C for 12 h and then grounded. The ionic liquid (IL) 1,5-diazabicyclo[4.3.0]non-5-ene-1-ium acetate ([DBNH]OAc) was synthesized from 1,5-diazabicyclo[4.3.0]non-5-ene (Fluorochem, UK) and acetic acid glacial (Merck, Germany), as described elsewhere.^15^ As the chicken feather in this study contained more than ∼90% keratin,^16^ it is used interchangeably with keratin (ker) in this report.

The keratin–cellulose blend solution was prepared by gradually adding the keratin powder into the IL at 90 °C. The solution was hand-mixed thoroughly and then was stirred mechanically at 30 rpm under vacuum (10–20 mbar) for 70 min at 90 °C to assure almost quantitative dissolution of keratin. The cellulose pulp was subsequently added into the keratin–IL solution and the resulting blend-solution was continuously mixed for 90 min. To investigate the effect of keratin on the spinning process, three initial shares of keratin [10, 25, and 50% (w/w) of the total polymer concentration] were used. The concentration of total polymer and the IL in the solution was fixed at 13% (w/w) and 87% (w/w), respectively. A reference cellulose solution (13% w/w) was prepared with a similar method as described previously.^15^

Due to the low-molecular weight of the keratin (∼10 kDa),^16^ a 100% keratin solution was not prepared because it does not have the viscoelastic properties required for dry-jet wet spinning. Solutions with a total polymer concentration of 15% w/w (initial keratin concentration of 25%) and 16% w/w (initial keratin concentration of 50%) were prepared to adjust the viscoelastic properties that decreased as the keratin share increased in the solutions.

The viscoelastic properties of the keratin–cellulose spinning solutions were measured by Anton Paar Physica MCR 302 rheometers (Austria). A dynamic frequency sweep test (100–0.1 s^–1^) was performed to measure the complex viscosity η* and dynamic moduli (storage modulus *G*′ and loss modulus *G*″) as a function of angular frequency, *ω*. The apparent zero shear viscosity *η*_0_* was derived from the Carreau-Gahleithner model, assuming both cellulose and keratin–cellulose solutions follow the Cox-Merz rule.^17,18^

The spinning solutions were then spun via a dry jet-wet spinning unit (Fourné Polymertechnik, Germany) termed Ioncell.^15^ The take-up velocity and extrusion velocity were adjusted so that the fibers were spun at a draw ratio (DR) of 4 as an optimum DR for achieving a highly oriented cellulose structure and sufficient diameter to compensate for the mass loss during the pyrolysis process.^10^ Lastly, the collected continuous filaments were washed (65–70 °C) and then air-dried.

### Preparation of CFs Derived from Keratin–Cellulose Composite Fibers

Up to 300 mg of the oven-dried composite fiber (∼10 cm length) was placed in a ceramic boat and pyrolyzed in a tubular furnace (NBD furnace) at a constant N_2_ gas flow (0.5 L/min). The temperature of the tubular furnace was raised from room temperature to the final temperature (500, 700, or 900 °C) at 10 °C/min heating rate, and then held for 30 min. The yield of CF is calculated by eq 1.
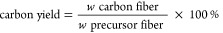
1

### Characterization of Composite and CFs

Thermogravimetric analysis was performed on a STA 449 F3 (Netzsch, Germany) and Hitachi STA7200RV under an inert atmosphere. The measurements were carried out from room temperature until 900 °C at 10 °C/min heating rate and an initial weight sample of 5–10 mg.

The mechanical properties of the composite fiber (linear density, tenacity, and modulus) were measured by a Favigraph tensile tester (Textechno). The measurement was carried out in the conditioned state (20 ± 2 °C and 65 ± 2 RH) with a gauge length of 20 mm and a testing speed of 20 mm/min. The values were averaged from 20 individual fibers.

The measurement of the total orientation of the composite fibers was conducted in a polarized light microscope (Zeiss Axio Scope with a 5λ Berek compensator). The birefringence (Δ*n*) was acquired by dividing the retardation of the polarized light by the fiber thickness, the latter being calculated from the linear density assuming a fiber density of 1.5 g/cm^3^. Total orientation factor *f*_t_ was calculated from the division of the birefringence by the maximum value of birefringence of cellulose (0.062).^19^

The chemical functionalities of the composite and CFs were investigated by attenuated total reflection–Fourier transform infrared spectroscopy (ATR–FTIR) Nicolet6700 with 64 scans, a 4 cm^–1^ resolution, and a wavenumber range of 4000–650 cm^–1^.

Elemental analysis of the composite fiber and CFs was measured by a Perkin Elmer 2400 CHNS/O Analyzer and a Thermo Fisher EA 1108. The C, H, and N (wt %) content was directly obtained from the measurement, while the O (wt %) was calculated from the mass difference. Each sample was measured in duplicate.

Scanning electron microscopy (SEM) images of the composite and CFs were acquired using a Zeiss Sigma VP with secondary electron detector. The composite fibers were broken by means of cryo-fracture while the CFs could be pulled apart manually.^10^ The samples were sputtered by gold/palladium prior to measurement. The imaging of the precursor was done at 3 kV while the CF was at 3–5 kV.

Block-face SEM imaging was performed to visualize the distribution of keratin in the cross sections of the composite fibers. Bundles of composite fibers with different keratin concentrations were immersed in 1 wt % OsO_4_ aqueous solution for an hour. They were subsequently washed with water, dehydrated through ethanol series and propylene oxide, and embedded in an epoxy resin (Embed 812, Electron Microscopy Sciences). The cross sections of the fibers were exposed on the block face using a 45° diamond knife (Diatom) equipped on an ultramicrotome (Leica EM UC6, Leica Microsystems). The SEM observation was carried out with field-emission SEM (Quanta-FEG 250, Thermo Fisher Scientific Inc.) operated at 10 kV and using a concentric backscattering electron detector.

X-ray diffraction data collection, processing, and analysis were described in detail in the Supporting Information. Wide-angle X-ray diffraction (WAXD) data of the powdered samples were collected in a transmission mode setting of a CuKα X-ray instrument, SmartLab (RIGAKU) operated at 45 kV and 200 mA.^10^ The collected data were calibrated for air scattering, sample holder scattering, and inelastic scattering. The crystallinity (CI) was estimated by a background subtraction method. The amorphous background corrected data of composite and keratin powder were used to subtract the scattering contribution of keratin in the composite fibers. After the subtraction of the keratin contribution, crystal widths (CW) were obtained from the Scherrer equation after a curve fitting procedure. The azimuthal intensity profile was obtained from the crystallographic (020) lattice plane (22.1° by 2*θ*) and was used to estimate the Hermans orientation parameter between the fibril axis and crystallographic *c* axis (*f*_WAXD_).

Small-angle X-ray scattering (SAXS) data were collected in a transmission mode of Xeuss 3.0 (Xenocs) CuKα X-ray operated at 50 kV and 0.6 mA. The data were processed correcting for the cosmic background, detector orientation, acquisition time, and transmitted flux. The orientation distribution of the samples was estimated from the equatorial streak. The data processing was carried out using XSACT software (Xenocs).

The Raman spectra of the CF were acquired using a LabRAM HR (HORIBA) equipped with a CCD detector. The measurement was done using a 514 nm Ar laser for 60 s at 0.1% laser power (14 μW on the sample) and at 100× microscope objective. The data processing was described previously in detail.^11^ The peak at 1350 cm^–1^ (D band) was fitted with a Lorentzian function, whereas the peak at 1590 cm^–1^ (G Band) with a Breit–Wigner–Fano (BWF) function.^20,21^ The *I*_D_/*I*_G_ ratio was calculated from the height of the two peaks.

The electrical conductivity [*σ* (S cm^–1^)] of the CF was calculated from the electrical resistance [*R* (Ω)] using eq 2. *l* refers to the fiber length between the two probes (cm) and *A* is the cross-sectional area of the fiber (cm^2^). The diameter of the CF was determined using an optical microscope (Zeiss, Germany). The resistance was measured by a multimeter with two-point probe (Agilent 4154A). The reported value for each sample was an average from 10 specimens.
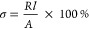
2

## Results and Discussion

### Spinning of Keratin–Cellulose Composite Fibers

**Figure 1:**
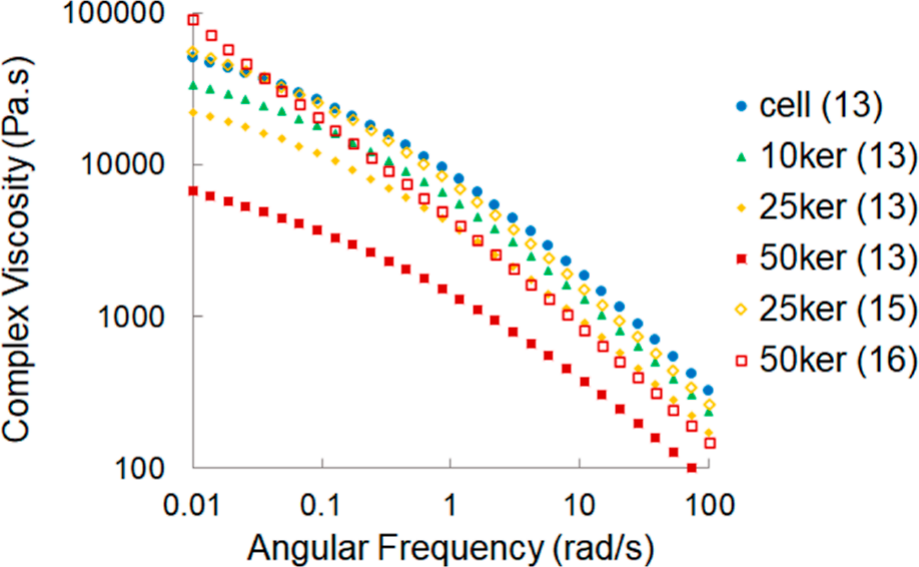
Complex viscosity of the spinning dopes of pulp cellulose mixed with 0, 10, 25, and 50 wt % of initial keratin concentration at 70 °C. The dopes had a total polymer concentration of 13, 15, or 16 wt % in [DBNH]OAc.

The complex viscosities of the spinning dopes obtained through frequency sweep measurements are shown in Figure 1. The keratin–cellulose dopes demonstrated a shear thinning-type behavior meaning that the complex viscosity decreases with increasing angular frequency. Although the measurements did not extend to the actual plateau value at a low angular frequency, the curves show a clear concave shape, which is typical for non-cross-linked polymer melts and solutions.^22^ Fitting and extrapolating the data sets with a Carreau-Gahleitner function gave plateau values, which are assumed to be identical to the zero shear viscosities. A higher keratin and lower cellulose content in the spinning dopes led to a decrease in the complex viscosity due to the much lower molar mass of the dissolved keratin (ca. 10 kDa as compared to ca. 260 kDa for cellulose). This effect is also observed with the dynamic moduli (Figure S1, Supporting Information), where the higher share of keratin shifted the crossover point (*G*′ = *G*″) of the spinning solutions to the lower modulus value and higher angular frequency or lower relaxation time.^22,23^ The drop in the crossover modulus and relaxation time implies reduced entanglements and interactions between the polymers because of the lower amount of relatively long cellulose chains.^18,24^

A similar dilution effect was observed in other studies when using low-molecular-weight co-biopolymers such as lignin and chitosan.^10,23^ To compensate for the decrease in the viscoelastic properties, spinning of the keratin-containing solution at the 13 wt % total polymer concentration was carried out at lower temperatures than the standard cellulose solution, as shown in Table 1. A higher total polymer concentration, and thus a higher loading of cellulose at a constant volume, could also improve the viscoelastic properties of the spin solutions.^23^ This is shown by the significantly higher zero shear viscosity of dope with 15 wt % total polymer concentration, and thus a higher spinning temperature, compared to that at 13 wt % (Table 1). At 16 wt %, the complex viscosity did not follow a concave trend anymore and instead showed a power-law behavior within the measurement range.^23^ Such a power-law dependence and the absence of a crossover point (of the dynamic moduli, Figure S1) indicate a strong gel character or crosslinked polymer system and does not allow to extract a plateau value for the viscosity through extrapolation. Spinning is usually possible if the solution has a certain viscoelasticity that is a defined balance in viscous and elastic properties.^25^ However, also solutions with a behavior resembling the 16 wt % solution reported herein were spun successfully.^26^

**Table 1:** Rheological Parameter of the Spinning Dopes of the Pulp Cellulose and Mixture of the Pulp and Feather Keratin at Different Concentrations Dissolved in [DBNH]OAc

dope sample	initial ker^a^ conc. (wt %)	total conc. (wt %)	*T* (°C)	*η*_0_^b^ (kPa s)	ω^c^ (1/s)	*G*′ = *G*″^d^ (Pa)
cell (13)		13	82	25.4	1.6	4100
10ker (13)	10	13	78	47.5	0.7	3100
25ker (13)	25	13	68	52.2	0.5	2200
50ker (13)	50	13	66	16.0	1.3	1150
25ker (15)	25	15	81	65.3	0.6	3100
50ker (16)	50	16	70			

a ker = feather keratin.

b *η*_0_ = zero shear viscosity.

c *ω* = angular frequency at a crossover point.

d Modulus at a crossover point.

### Effect of Keratin Incorporation in the Composite Fiber

#### Elemental Analysis of the Composite Fiber

Table 2 shows the elemental content of the cellulose and composite fibers measured by elemental analysis. Overall, a higher initial concentration of keratin in the spinning dope led to a higher nitrogen content (N/C) in the composite fibers, similar to a previous study with chitosan.^10^ However, the actual incorporation was generally lower (∼50%) than the keratin concentration in the initial dope, indicating a considerable keratin loss during the spinning process. The loss was attributed to the degradation of keratin induced by the elevated temperature and long processing time during dissolution and spinning.^27−29^ A notable amount of water-soluble amino acids and polypeptides dissolved in the coagulation bath or continuous washing line.^30−32^ This is also shown in the almost undetectable sulfur content (S/C) in the resulting composite fibers, suggesting the release and loss of almost all sulfur-containing compounds during the spinning process.

**Table 2:** Carbon (C) Content (wt %) and Other Elements (mol/mol) and the Estimated Actual Keratin Incorporation (wt %) in the Composite Fibers at Different Initial Keratin Addition and Total Polymer Concentrations

		elemental content (mol/mol)					incorporated ker conc (wt %)	
sample	C (wt %)	H/C	N/C	S/C	O/C	intended ker^a^ conc. (wt %)	based on N/C	based on literature^b^
cell (13)	42.6	1.80	0.00	0.00	0.90			
10ker (13)	43.8	1.77	0.01	0.00	0.84	10.0	3.4	4.1
25ker (13)	44.8	1.78	0.03	0.00	0.78	25.0	13.2	13.1
50ker (13)	45.4	1.78	0.07	0.00	0.72	50.0	28.0	26.9
25ker (15)	42.9	1.60	0.03	0.00	0.87	25.0	10.3	10.1
50ker (16)	46.3	1.73	0.08	0.00	0.68	50.0	31.4	30.7
ker powder	51.7	1.62	0.26	0.02	0.34			

a ker = feather keratin.

b According to the calculation in Nuutinen et al.^28^

#### Structural and Mechanical Properties of the Keratin–Cellulose Composite Fibers

Table 3 shows the structural parameters of the cellulose and the composite fibers with varying keratin concentrations. The total polymer concentration in the spinning solution did not influence the structural parameters of the respective composite fibers. The differences were within the statistical error range.

**Table 3:** Structural Parameters of the Cellulose and Keratin-Containing Composite Fibers from WAXD, SAXS, and Birefringence

		WAXD			SAXS	birefringence
sample	actual ker conc^a^	CW (Å)	CI (%)	*f*_WAXD_	microvoid orientation	total orientation
cell (13)		34.4 ± 1.4	35.2 ± 2.0	0.82 ± 0.02	0.89 ± 0.02	0.70 ± 0.05
10ker (13)	3.4	33.7 ± 0.9	35.3 ± 1.1	0.81 ± 0.05	0.88 ± 0.01	0.65 ± 0.10
25ker (13)	13.2	33.6 ± 0.6	33.1 ± 1.0	0.82 ± 0.02	0.80 ± 0.01	0.62 ± 0.05
50ker (13)	28.0	34.1 ± 1.3	30.3 ± 1.6	0.78 ± 0.05	0.83 ± 0.00	0.46 ± 0.06
25ker (15)	10.3	33.7 ± 1.0	34.1 ± 0.4	0.80 ± 0.01	0.84 ± 0.00	0.61 ± 0.05
50ker (16)	31.4	33.1 ± 2.2	29.4 ± 1.2	0.81 ± 0.01	0.80 ± 0.01	0.49 ± 0.05

a Actual ker conc = incorporated keratin concentration based on EA.

The cellulose crystal size in the composite fibers and crystalline orientation parameters (*f*_WAXD_) remained unaffected by the addition of keratin. However, fiber crystallinity, microvoid orientation, and total orientation showed changes depending on the share of keratin.

The composite fiber with the lowest keratin content 10ker (10 wt % initial and ∼4 wt % actual concentration) showed a similar fiber crystallinity and total polymer orientation to that of the reference cellulose fiber cell. In addition, the microvoid orientation determined via SAXS of 10ker and cell were almost identical, indicating an unaffected organization of the fibrillar network surrounding the microvoids. Regenerated cellulose fibers are constructed by a multi-level hierarchical structure,^33^ which includes elementary fibrils consisting of crystalline and amorphous regions,^34^ and microvoids in between the fibrils.^35^ The unaltered structure and organization of the cellulose fibrils suggest a preserved hierarchical structure of the cellulose fibrillar network at a low actual keratin concentration. At 25 wt % addition (∼12 wt % actual conc.), the fiber crystallinity was roughly constant, whereas the microvoid orientation in the composite fiber decreased notably, indicating an altered cellulose fibrillar network. The total orientation of the composite fiber was also lower than the cellulose fiber, although the value was within a standard deviation. At an addition of 50 wt % (∼30 wt % actual conc), the composite fiber 50ker exhibited a significantly lower value of the fiber crystallinity, microvoid orientation, and total orientation than the cellulose fiber, suggesting a disturbed cellulose structure and less organized fibrillar network in the composite fiber at high levels of keratin incorporation. The disturbed cellulose structure, particularly at a high keratin concentration, is likely due to the formation of the phase-separated keratin agglomerates in the composite fibers. At low keratin content, up to 12 wt % actual concentration, the cellulose matrix is mostly uncompromised with sporadically distributed small agglomerates. The crystallinity is thus unaffected although a drop in total orientation is visible. At a larger keratin content, the cellulose matrix is interspersed with protein phases, which also affects the total crystallinity of the fiber. The morphology is explained further in the next chapter.

Table 4 shows the mechanical properties of all fibers spun at a draw ratio of 4. An earlier study showed that the tenacity of cellulose fibers spun via the Ioncell process depends on the cellulose concentration of the spinning solution.^37^ Such a trend was not observed here. This was attributed to two reasons. First, the fibers were spun at a moderate draw ratio of 4, where the differences in mechanical properties are less pronounced. Second, due to the loss of keratin, the actual difference in the total polymer concentration was relatively small.

**Table 4:** Mechanical Properties of the Cellulose and Keratin-Containing Composite Fibers

sample	actual ker conc. (wt %)	tenacity (cN/tex)	Young’s modulus (GPa)	elongation (%)	diameter, μm^a^
cell (13)		42.9 ± 3.2	15.7 ± 1.0	12.0 ± 1.0	17.5 ± 2.8
10ker (13)	3.4	38.4 ± 2.6	15.6 ± 1.1	11.0 ± 1.1	16.6 ± 2.8
25ker (13)	13.2	41.2 ± 1.9	16.4 ± 1.0	9.9 ± 1.0	16.8 ± 1.2
50ker (13)	28.0	32.4 ± 1.8	13.9 ± 0.8	9.1 ± 0.8	15.2 ± 1.4
25ker (15)	10.3	35.9 ± 1.7	14.7 ± 1.1	11.5 ± 1.0	16.9 ± 1.3
50ker (16)	31.4	31.3 ± 1.7	12.6 ± 0.9	10.6 ± 1.4	17.2 ± 1.6

a The diameter is calculated from the linear density assuming a fiber density of 1.5 g/cm^3^.^36^ For fibers with higher keratin content, the actual density might be slightly different.

Based on empirical studies, Krässig has postulated that the tenacity of man-made cellulose fibers in the conditioned state is proportional to (1/DP_*nL*_ – 1/DP_*n*_)·CrI·*f*_t_^2^, where DP_*nL*_ is the length of the crystallites, DP_*n*_ is the cellulose DP, CrI is the degree of crystallinity, and *f*_t_^2^ is the square of the degree of orientation.^37,38^ The addition of minor amounts of keratin did not affect much the degree of crystallinity and orientation, respectively. This is reflected in similar tenacity values of cell, 10ker, and 25ker spun at the 13 wt % total polymer concentration. At a higher keratin concentration (50ker), the structural parameter decreased, which is one reason for the drop in tenacity and Young’s modulus. The increase in number and size of keratin agglomerates certainly plays a role too. This will be discussed in a later section.

A similar reduction in tenacity was found in cellulose–lignin composite fibers.^23,39^ At ∼ 30 wt % actual lignin content, the fiber tenacity dropped from ca. 45 cN/tex of the pure cellulose fiber to ca. 26 cN/tex.^39^ Lignin is also incompatible with cellulose and forms separated domains. However, lignin remains more evenly distributed in the fiber, which might explain the slightly higher drop in mechanical properties as it affects the entire cellulose matrix.

#### Effect of Keratin on the Char Yield and Thermal Degradation Properties of the Composite Fiber

**Figure 2:**
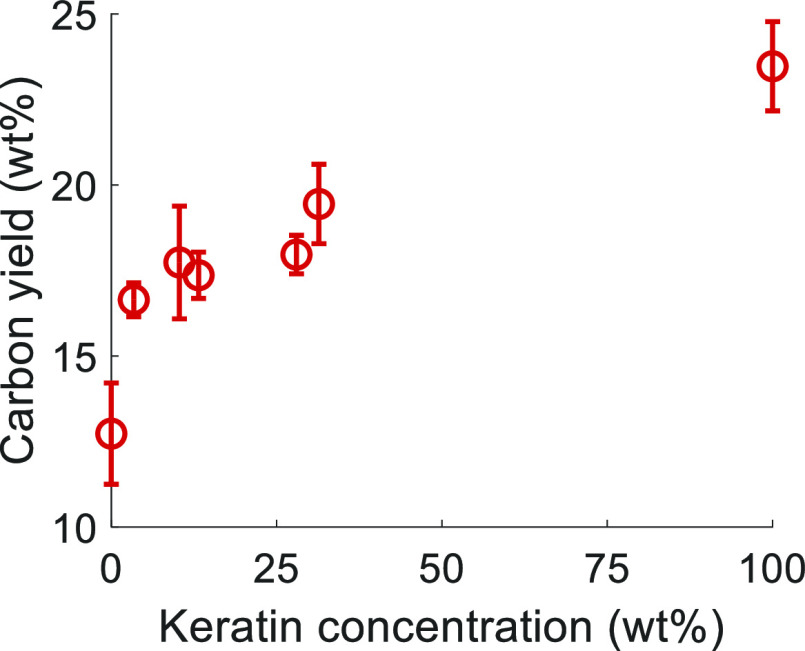
Carbon yield (wt %) of cellulose and composite fibers with different actual keratin incorporation in the fibers (wt %) measured via thermogravimetric analysis (TGA).

Figure 2 shows the carbon yield of the composite fibers with different keratin concentrations after pyrolysis during TGA until 900 °C. The actual keratin incorporation from ∼4 to ∼30 wt % increased the carbon yield of the cellulose fiber by ∼30 to 53%, respectively. At the same actual incorporation in the fiber (∼30 wt %), keratin produced a comparable yield increment with organosolv lignin, although the carbon yield from lignin powder (∼31%) was higher than keratin powder (∼23%).^23^ The increase in carbon yield from lignin was mainly connected to its originally high carbon content and the highly cross-linked polyphenolic structures.^40,41^ By contrast, keratin seems to interact with the cellulose matrix during pyrolysis, showing some catalytic activity similar to chitosan.^10,11^

The experimental carbon yield of the composite fibers was compared with the anticipated carbon yield in the absence of any polymer interaction during pyrolysis. The anticipated TG curve and final carbon yield were calculated through the weighted mean of the neat cellulose fiber and keratin powder according to eq 3.^42^
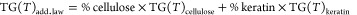
3
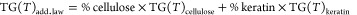

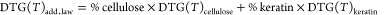
4

The experimental and calculated TG curves can be seen in Figure S2. The experimental and calculated carbon yields are tabulated in Table 5. For all composite fibers, the carbon yield obtained from the experiment was ∼20% higher than the anticipated values, suggesting a moderate catalytic effect of keratin on enhancing the carbon yield during cellulose pyrolysis.

**Table 5:** Comparison of the Experimental and Calculated Carbon Yields

		wt % after carbonization at 900 °C		
samples	incorporated ker (wt %)	experimental	calculated via add. law^a^	ratio exp./calc.
cell (13)	0	12.7 ± 1.5	12.7	
10ker (13)	3.4	16.6 ± 0.5	13.1	1.27
25ker (13)	13.2	17.4 ± 0.7	14.2	1.23
50ker (13)	28.0	18.0 ± 0.6	15.8	1.14
25ker (15)	10.3	17.7 ± 1.6	13.8	1.28
50ker (16)	31.4	19.4 ± 1.2	16.1	1.20
keratin powder	100	23.5 ± 1.3	23.5	

a Based on the carbon yield of cell (13) fibers and keratin powder.

**Figure 3:**
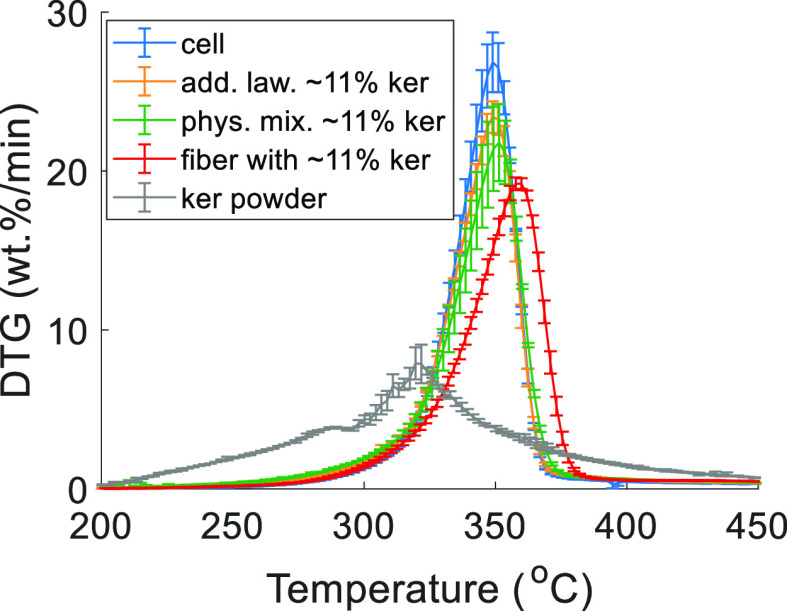
Comparison of DTG curves of the powdered cellulose fiber, powdered composite fiber containing ∼11 wt % ker [25ker (15)], the physical mixture of powdered cellulose fiber and ∼11 wt % ker powder, and calculated curve at ∼11 wt % ker addition. Error bars represent a standard deviation from different samples.

Further, the experimental DTG curve of the composite fiber was compared with the anticipated DTG curve calculated in a similar manner using eq 4, plotted in Figure 3. The composite fiber had a slightly lower peak area of cellulose degradation than the calculated DTG curves with respect to the pure cellulose fiber (∼89 and ∼97%, respectively). In addition, there was a slight shift in the peak maxima to a higher temperature compared to the cellulose reference. A similar shift of the thermal degradation peak to higher temperatures and an increase in the char yield was also shown for biomass pyrolysis with CaO as the catalyst.^43^

The DTG curve of the composite fiber containing ∼11 wt % keratin [25ker (15) fiber] was also compared to a physical mixture of powdered cellulose fiber with 11 wt % keratin powder (Figure 3). Powdered cellulose fiber was used in the physical mixture, instead of the original cellulose pulp, to exempt possible effects of residual IL^44,45^ or the cellulose polymorph^46^ in the regenerated cellulose fibers on thermal degradation behavior. The composite fiber had a lower peak area of cellulose degradation compared to the physical mixture (∼89 and ∼96%, respectively). The shape of degradation curve was also different from the physical mixture. The latter, on the other hand, had a similar shape and very close peak area of cellulose degradation of the calculated curve, indicating a lack of interaction between the cellulose and keratin when in mere physical contact. The close contact of keratin with cellulose in the composite fiber matrix enables the catalytic activity of the amine and amide groups present in keratin, as previously shown for chitosan–cellulose composite fibers.^10,11^Figure S3 shows FTIR spectra of cellulose, keratin, and the composite fibers. The amide II band at ∼1550 cm^–1^ is clearly visible in composite spectra.^47−49^

**Figure 4:**
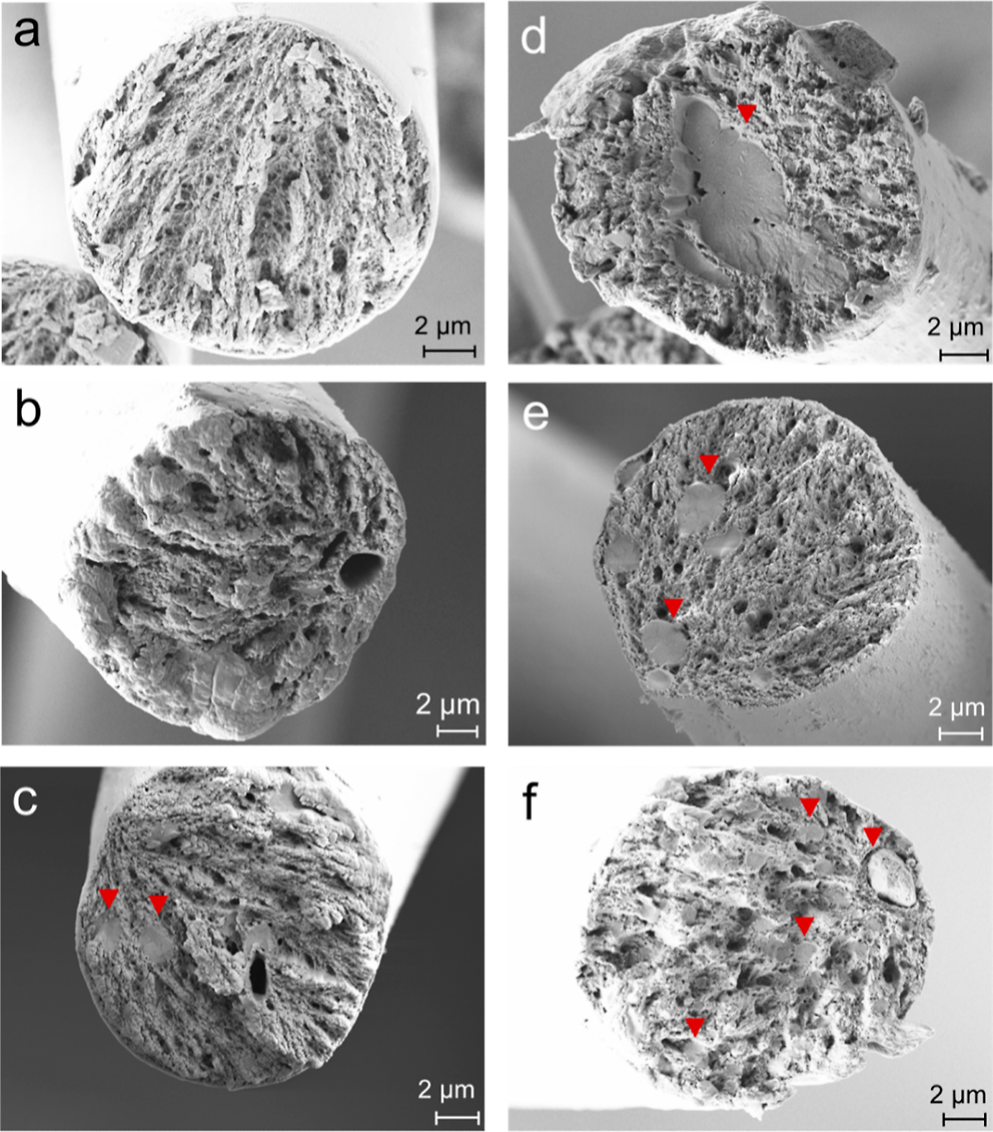
SEM of cross-section images of the composite fibers of cellulose (a), 10ker (13) (b), 25ker (13) (c), 50ker (13) (d), 25ker (15) (e), and 50ker (16) (f) (ker refers to initial keratin in the dope solution). Red arrowheads point toward the keratin domains.

**Figure 5:**
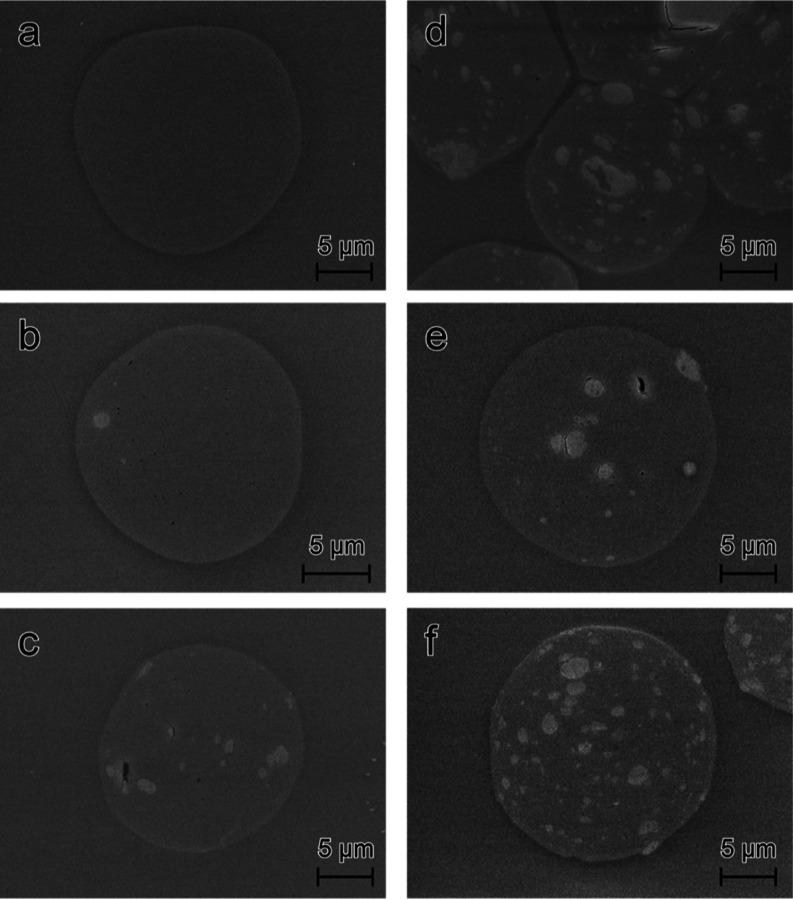
Block-face SEM images of cross sections of the composite fibers of cellulose (a), 10ker (13) (b), 25ker (13) (c), 50ker (13) (d), 25ker (15) (e), and 50ker (16) (f) (ker refers to initial keratin in the dope solution). Keratin domains are visible as white spots due to the preferential staining of keratin proteins with OsO_4_.

As indicated briefly earlier, the copolymeric fiber matrix did not appear homogeneous. The keratin used in this study dissolved well in [DBNH]OAc and formed a homogeneous solution in the presence of pulp cellulose. However, under coagulation conditions, keratin resumed a supramolecular structure that seems mostly incompatible with cellulose, leading to a heterogeneous fiber matrix. SEM images of the fractured surface and block-face SEM images using staining agents are shown in Figures 4 and 5, respectively (more images can be found in Supporting Information, Figures S6–S10). Two distinct phases are visible in all keratin-containing fibers. The size of the keratin domains varies from a few tens of nanometers to a few micrometers not only between the composite fibers with different keratin concentrations but also within single cross sections. In some cases, the cross sections were completely covered by keratin domains for both composite fibers with the lowest [10ker (13)] (Figure S6c,d) and the highest keratin content (50ker (16) (Figure S10b). In obliquely fractured fibers (Figure S6a and S9c), the keratin domains were observed elongated along the fiber axis. The observed polydispersity in both size and morphology of keratin domains makes it challenging to estimate the contact surface area between keratin and cellulose based on the SEM images.

Besides keratin agglomerates, cavities or open pores are clearly visible in the cross section of the keratin composite fibers (Figures 4 and S6–S10), which was possibly caused by leaching of the water-soluble keratin fraction out from the fibers. The presence of keratin agglomerates and micropores reduces the contact between the cellulose and keratin further and affect the resulting microvoid orientation, as listed in Table 3. This would explain why the catalytic activity of keratin in terms of carbon yield of the cellulose composite fibers was generally lower than that of chitosan.^10^ For the same initial total polymer concentration of 13 wt %, the catalytic effect shows a decreasing tendency with increasing keratin concentration (Table 5). This supports the observation that the contact between cellulose and keratin is not as intimate as in the case of cellulose chitosan composite fibers.

**Figure 6:**
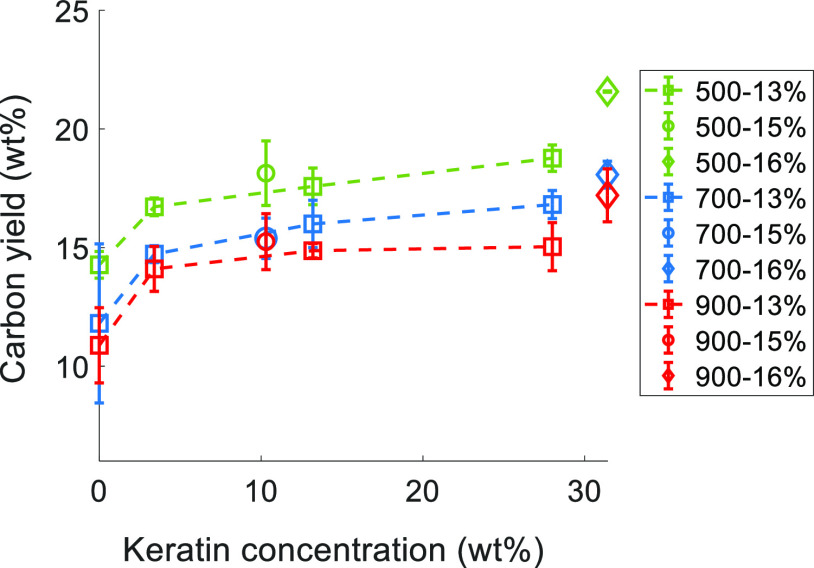
Carbon yield from oven pyrolysis of the composite fibers with different incorporated keratin concentrations and total polymer concentrations.

Figure 6 shows the char yield of pyrolysis carried out in a tubular furnace at different temperatures (500, 700, and 900 °C) at the same heating rate as used in the TGA experiments (10 °C/min) and without any prior stabilization phase. The oven pyrolysis trials allowed one to compare the intermediate yields at different temperatures and to follow the evolution of the CFs through elemental analysis (Table 6). In line with the TGA results, the char yield was increased by the presence of keratin. A rise in char yield was already observed at ∼ 4 wt % actual keratin content.

**Table 6:** Carbon Content (wt %) and Elemental Ratio (mol/mol) of Selected Precursor Fibers and CFs Obtained at Different Temperatures

	cell					25ker (15)				
temperature	C	H/C	N/C	S/C	O/C	C	H/C	N/C	S/C	O/C
no heat treatment	42.6	1.80	0.00	0.00	0.90	42.9	0.60	0.030	0.00	0.87
500	82.1	0.38	0.01	0.00	0.75	74.7	0.51	0.050	0.00	0.50
700	90.2	0.19	0.00	0.00	0.06	81.1	0.23	0.046	0.00	0.12
900	92.0	0.07	0.00	0.00	0.05	81.9	0.22	0.039	0.00	0.12

As expected, the final char yield decreased with increasing pyrolysis temperature (from 500 to 900 °C) due to the progressive release of volatile compounds such as H_2_O, CO, CO_2_, and C_*x*_H_*y*_O_*z*_ from the biomass matrix.^50^ In the course of the heat treatment, the residue turned into a carbonaceous structure, as shown by the increase in the carbon content (wt %) and a concomitant decrease in relative oxygen and hydrogen content (O/C and H/C, respectively) (Table 6). In the case of the pure cellulose precursor fiber, the oxygen and hydrogen content decreased continuously with rising heat treatment to 900 °C. However, for the keratin containing fibers, the oxygen content first dropped until 700 °C and then remained constant at 900 °C. Moreover, the hydrogen content of the keratin–cellulose-derived CFs was higher than for the cellulose-based CF at all the studied temperatures. This also confirms an altered pyrolysis mechanism in the case of the composite fibers.

Contrary to the evolution of the hydrogen and oxygen content in the composite-derived CFs, the nitrogen content (N/C) first increased at 500 °C and then slightly dropped at higher temperatures. However, the nitrogen content of the CF at 900 °C was still higher than in the original composite precursor fibers. This is in line with earlier studies showing that nitrogen remains incorporated in the carbon structure at moderate pyrolysis temperatures.^10^ The formation of volatile nitrogen-containing species requires pyrolysis temperature higher than 500 °C. Further, there seems to be a critical relative nitrogen content in the carbon matrix. The CF pyrolyzed at 900 °C from composites with up to ∼14 wt % keratin showed a higher N/C than the original precursor. By contrast, the fibers with a keratin content of ∼30 wt % [50ker (13) and 50ker (16)] yielded CFs with a lower N/C compared to the original composites (Table 7). Nevertheless, the N/C ratios of the CFs at 900 °C derived from composites with ∼30 wt % keratin were still higher than those from the lower keratin incorporation (≤14 wt %). This suggests that once a certain threshold nitrogen content is reached, the nitrogen is more readily released upon further heat treatment.

**Table 7:** Relative Elemental Composition (mol/mol) of the CFs Obtained at 900 °C Derived From Precursor Fibers with Different Keratin Contents

	composite fibers		CFs at 900 °C				
sample	keratin conc. (wt %)	N/C	C (wt %)	H/C	N/C	S/C	O/C
cell	0	0	92	0.07	bdl	0	0.05
10ker (13)	3.4	0.01	82.4	0.16	0.024	0	0.13
25ker (13)	13.2	0.036	81.1	0.14	0.045	0	0.12
50ker (13)	28	0.075	79.3	0.15	0.064	0	0.13
25ker (15)	10.3	0.029	81.9	0.22	0.039	0	0.12
50ker (16)	31.4	0.084	79.6	0.15	0.064	0	0.12

### Effect of Keratin on the Properties of the CF

#### Effect of Keratin on the Nanostructure of the CFs

**Figure 7:**
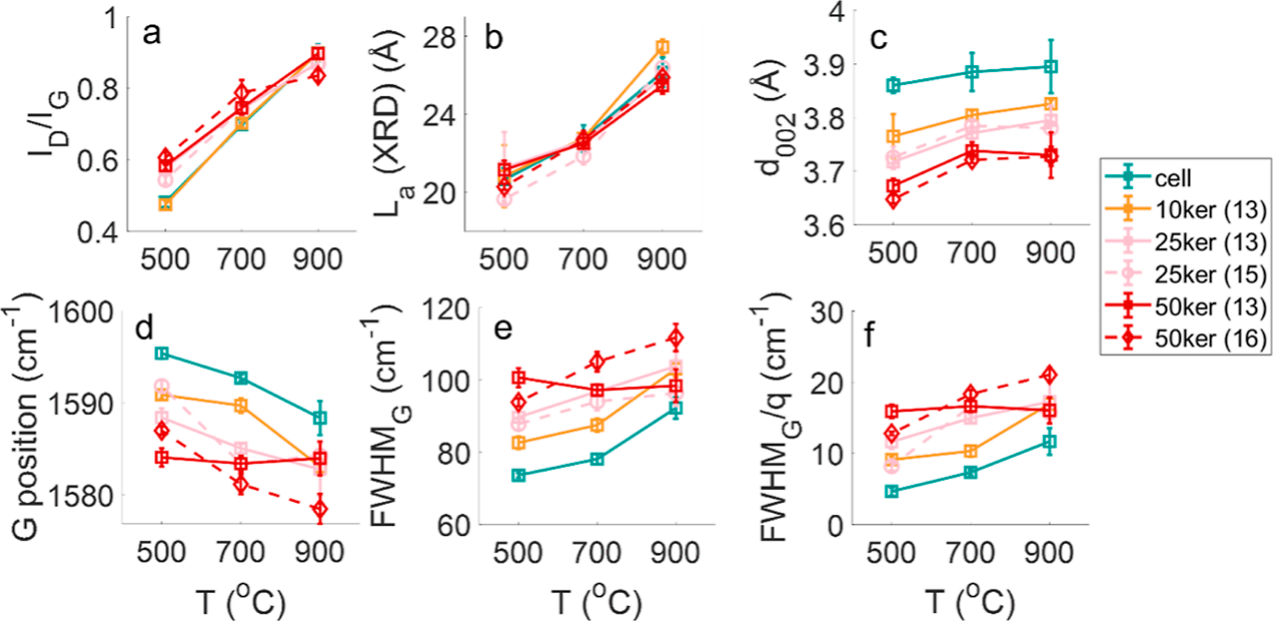
*I*_D_/*I*_G_ ratio (a), crystallite size *L*_a_ from X-ray diffraction (XRD) (b), apparent interlayer distance *d*_002_ (c), position of the G peak (d), FWHM_G_ (e), and FWHM_G_/*q* (f) of the CFs derived from cellulose and keratin composite fibers as a function of the pyrolysis temperature.

Figure 7 shows the effect of the pyrolysis temperature on the structural properties of the CF measured by Raman spectroscopy and XRD. Overall, the increase in the pyrolysis temperature from 500 to 900 °C increased the Raman parameter *I*_D_/*I*_G_ ratio in Figure 7a, which is attributed to the increase in the size of in-plane aromatic clusters.^5,51,52^ The extension of in-plane aromatic clusters shown by the rising *I*_D_/*I*_G_ is confirmed by the increase in the apparent crystallite size along the basal plane (*L*_a_) measured by XRD (Figure 7b). The simultaneous increase of the *I*_D_/*I*_G_ ratio and *L*_a_ induced by the heat treatment have been reported for other bio-carbons, such as those derived from cellulose,^5,51−53^ lignin,^53,54^ or mixed-polymer matrices like wood and bark.^55,56^

Besides the development of the size of the in-plane aromatic cluster, the XRD measurement showed a rise of the apparent interlayer distance of the aromatic planes *d*_002_, particularly from 500 to 700 °C (∼3.77 to 3.90 Å) (Figure 7c). The higher *d*_002_ could indicate a rising topological disorder of the in-plane aromatic cluster.^57,58^ Additionally, parameters extracted from the G band in Raman spectra, which include the peak position, full-width at half maximum (FWHM_G_), and FWHM_G_/*q* (*q* is the coupling coefficient in BFW fitting), can provide information about the in-plane ordering of the aromatic clusters. The G peak position is linked to the bond strength, and the FWHM_G_ is associated with the bond length and angle distortions of the in-plane aromatic clusters, while FWHM_G_/*q* approaching zero indicates the onset of the three-dimensional ordering of the basal plane.^20 ,21 ,59 −61^ An increase in the order of the in-plane sixfold aromatic clusters would be indicated by the shift of the G band position to a higher frequency and lower FWHM_G_ and FWHM_G_/*q* values.^20,60−62^ Hence, the downshift of the G peak and the increase in the FWHM_G_ and FWHM_G_/*q* in Figure 7d–f, respectively, particularly from 500 to 700 °C, reflect a growing disorder in the in-plane aromatic clusters.

A similar trend of the concurrent increase in the extension of in-plane clusters *L*_a_ with the rising topological disorders shown by the increase in *d*_002_ and the Raman ordering parameters at 500 to 700 °C were also observed for CFs derived from nitrogen-containing chitosan composite fibers.^11^ The presence of nitrogen, besides oxygen, in the precursor fibers can favor the formation of non-hexagonal rings (i.e., five-membered rings) as the energetically more favored route.^60,63−65^ Pentagonal rings can induce buckling of the planes and, hence, increase the topological disorders of the basal planes,^66−69^ reasonably explaining the increase in *d*_002_ in Figure 7c. The simultaneous increase of *L*_a_ and *d*_002_ at a relatively low pyrolysis temperature (<900 °C) was also reported for other oxygen-rich precursors such as wood^70^ and stabilized pitch fiber.^71^

**Figure 8:**
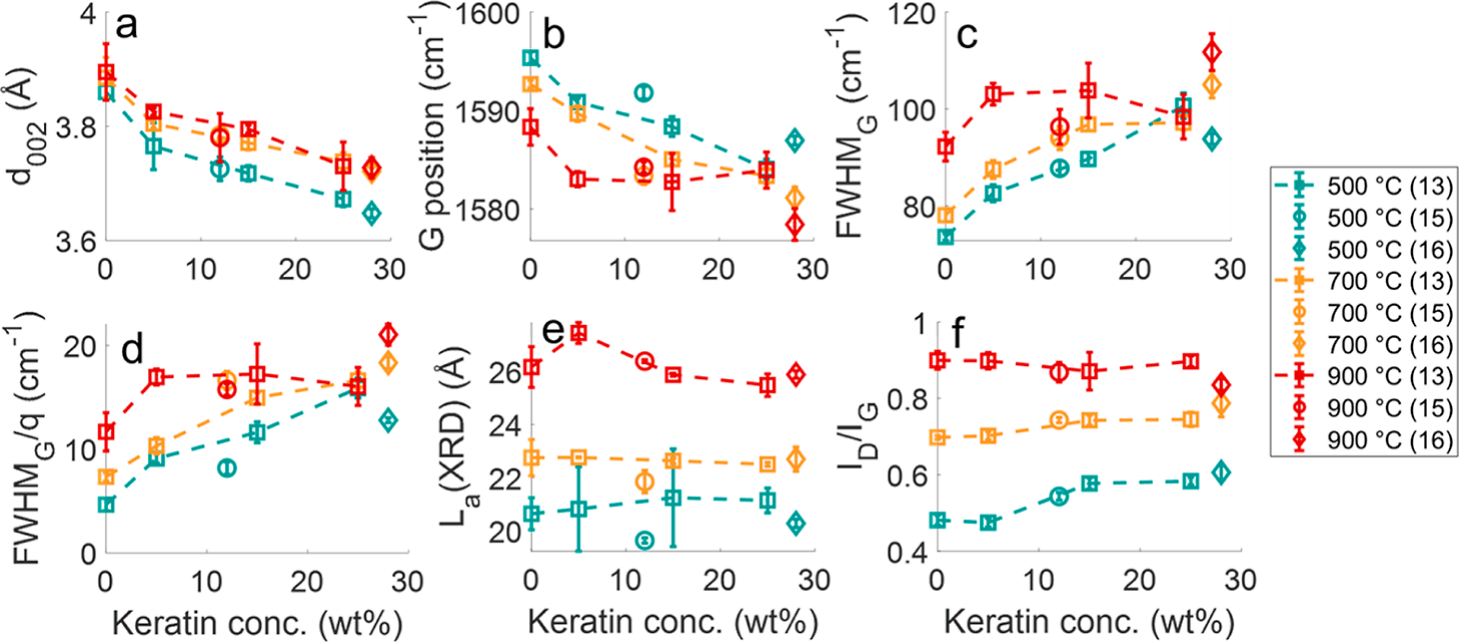
Apparent interlayer distance *d*_002_ (a), position of the G peak (b), FWHM_G_ (c), FWHM_G_/*q* (d), crystallite size *L*_a_ (e), and *I*_D_/*I*_G_ ratio (f) of the CFs as a function of keratin concentration in the respective composite fiber.

The effects of the incorporated nitrogen on the Raman and XRD parameters of the CFs were further studied by analyzing them against the actual keratin content of the precursor composite fiber (Figure 8). The *d*_002_ fell continuously with a higher keratin addition corresponding to an increased nitrogen incorporation in the carbon network (Figure 8a). However, there was a downshift of the G peak position and an increase in FWHM_G_ and FWHM_G_/*q*, suggesting an increase in the in-plane disorder (Figure 8b–d, respectively). These observations of a reduction in *d*_002_ along with the increase in the in-plane disorders suggested by the Raman G band parameters were also made for CFs derived from cellulose–chitosan composite fibers.^11^ The reduced *d*_002_ was not due to the flattening of the basal planes^58^ but was likely due to cross-link formation induced by the nitrogen inclusion in the carbon network.^69,72−76^ While the effect of residual nitrogen from keratin on *d*_002_ was markedly, the effect on *L*_a_ and the *I*_D_/*I*_G_ ratio was faint, as shown in Figures 8e,f, respectively. The absolute values of *L*_a_ and the *I*_D_/*I*_G_ ratio in this study were in the same range as those found for carbons derived from cellulose–chitosan composite fibers.^11^

#### Effect of Keratin on the Electrical Conductivity of the CFs

**Figure 9:**
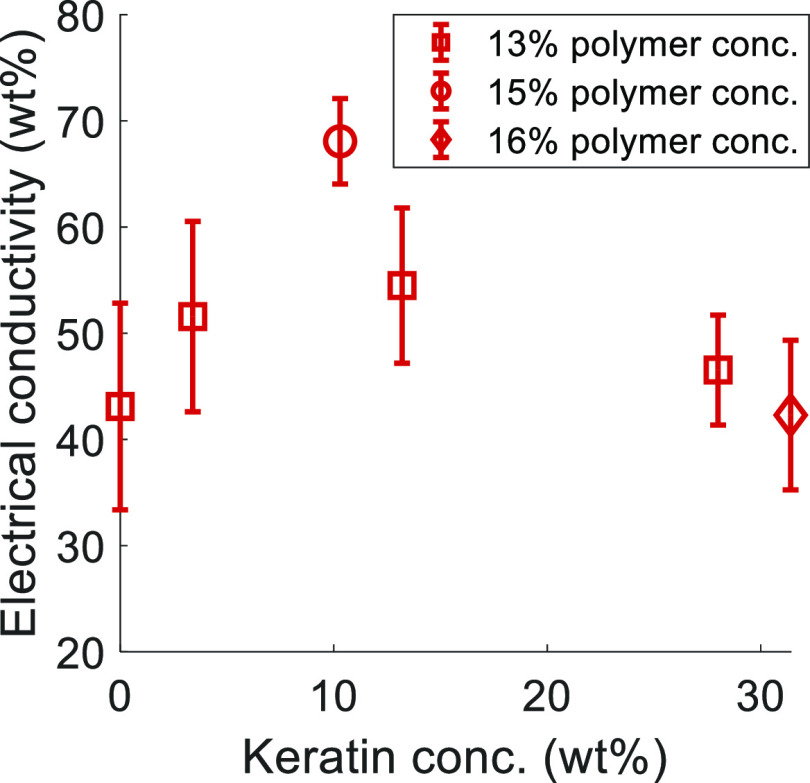
Electrical conductivity of CFs produced at 900 °C from precursor fibers with different keratin contents.

**Figure 10:**
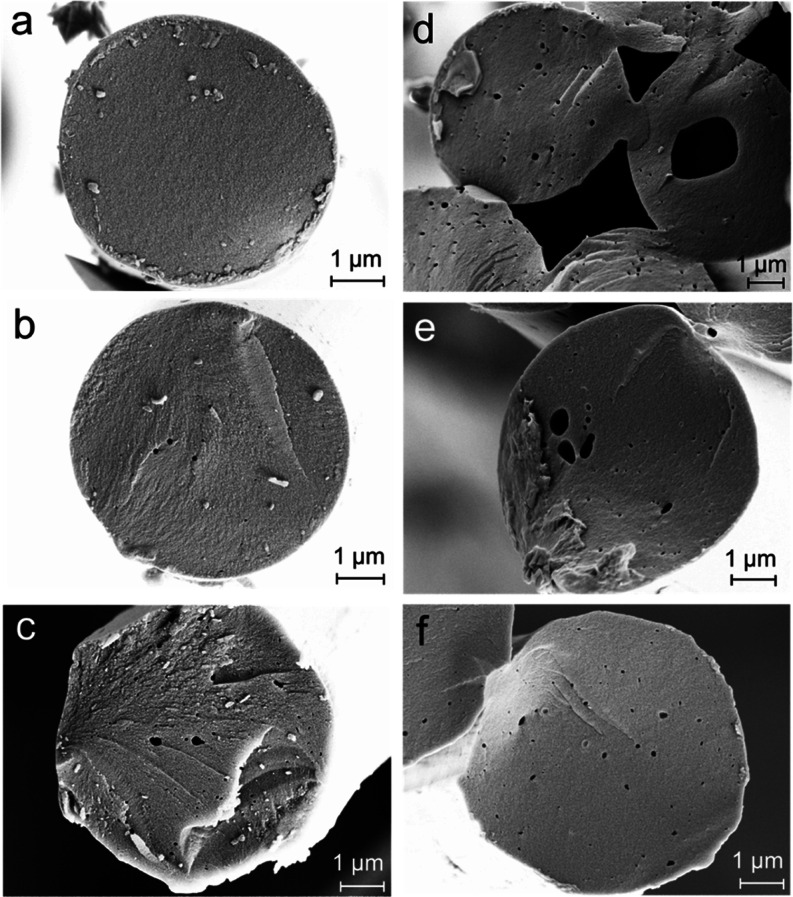
SEM images of CF cross sections pyrolyzed at 900 °C derived from cellulose (a), 10ker (13) (b), 25ker (13) (c), 50ker (13) (d), 25ker (15) (e), and 50ker (16) (f) (ker refers to initial keratin in the dope solution).

The gradual rise of the keratin content in the precursor fiber seemed to first increase the electrical conductivity of the respective CFs obtained at 900 °C, before further keratin addition led to a drop of the electrical conductivity (Figure 9). The highest value of ∼68 S/cm was achieved with the 25ker (15) CF. This was lower than the electrical conductivity reported for soy hull-derived carbon (∼120 S/cm)^77^ but much higher than the carbon from plywood and particleboard (<0.3 S/cm), both pyrolyzed under similar conditions.^78^ The influence of nitrogen incorporated in the carbon structure on the bulk conductivity of the carbon material has been studied for many different systems.^79−82^ However, the effect depends strongly on the underlying carbon matrix, the distribution and modification of the incorporated nitrogen, and the methods used to prepare those nitrogen-doped carbon scaffolds. Further, comparison with literature value was hampered due to the relatively porous morphology of the CFs derived from keratin-containing precursor fibers, as an increase in pores reduces the electrical conductivity of the carbon.^83,84^ The formation of the pores or large voids in the keratin–cellulose-derived carbon, which were also observed in the mixed-polymer precursor fibers, is shown in the SEM images in Figure 10. The CFs derived from standard cellulose fibers, on the other hand, showed a compact and dense structure, as reported previously.^10^ Further SEM cross-section images of the CFs, including the pores observed in the keratin composite fibers and the higher magnification images, can be found in Supporting Information Figures S11–S16.

These voids and pronounced porous structure are also suspected to be responsible for the observed brittleness of the CFs,^85,86^ despite the smaller interlayer spacing *d*_002_ found for carbons derived from keratin composite fibers. Previous studies reported a correlation between smaller interlayer spacing *d*_002_ and an improvement of the mechanical properties of the CFs,^87,88^ including the CF derived from the chitosan-cellulose composite fiber.^11^ The brittleness of the CFs prevented reliable mechanical testing of the CFs.

## Conclusions

This study investigated the potential of keratin derived from chicken feathers to act as a natural charring agent that can improve the yield of cellulose-derived CF. A small catalytic effect on enhancing the carbon yield was observed, albeit not as significant as seen earlier with other biopolymers such as chitosan. At low keratin concentrations the mechanical properties of the cellulose fibers remained largely unaffected. However, somehow surprisingly, it was found that keratin and cellulose phase separated during coagulation, leading to a discontinuous fiber structure with distinct keratin domains embedded in the cellulose matrix. Thus, chemical and structural compatibility with cellulose should be considered when utilizing a co-biopolymer to alter the properties of cellulose-based CFs. Upon carbonization, the structural parameters of the resulting CFs mostly depended on the carbonization temperature. The size of the in-plane aromatic clusters and crystallite size along the basal plane increased steadily from 500 to 900 °C. A topological disorder observed with increasing keratin content can be attributed to the incorporation of nitrogen in the carbon structure which introduces non-hexagonal rings into the carbon matrix. The mechanical properties of the CFs obtained through heat treatment of keratin–cellulose blend fibers at moderate temperatures up to 900 °C were only modest for structural applications. However, the presence of nitrogen and pores or voids in the carbon matrix can be beneficial for other applications that involve chemical or electrochemical processes. Such an application would require a further development of the carbonization protocol which also considers the pore size distribution and carbon matrix morphology.

## Supporting Information Available

Method of characterization of precursor fibers using WAXD and SAXS; dynamic moduli of spinning dopes; mechanical properties of composite fibers at DR 4 in the wet state; FTIR spectra of keratin-containing composite fibers; XRD fitting of the CF; Raman fitting of the CF; SEM images of keratin–cellulose precursor fibers; and SEM images of keratin–cellulose CFs (PDF)
