# Supplementary material for: Evaluation of Keratin–Cellulose Blend Fibers as Precursors for Carbon Fibers
Source: ACS Sustain Chem Eng. 2022 Jun 22;10(26):8314–25. doi: 10.1021/acssuschemeng.2c00976 (PMC9275789; doi:10.1021/acssuschemeng.2c00976)
Supplement: Supplementary file 1 — sc2c00976_si_001.pdf [file sc2c00976_si_001.pdf]

Supporting information

Evaluation of keratin-cellulose blend fibers as precursor for carbon fibers

Hilda Zahra [a], Julian Selinger [a, b], Daisuke Sawada [a], Yu Ogawa [c], Hannes Orelma [d], Yibo Ma [a], Shogo Kumagai [e, f], Toshiaki Yoshioka [e], Michael Hummel [a, *]

[a] Department of Bioproducts and Biosystems, Aalto University, Vuorimiehentie 1, 02150 Espoo, Finland

[b] Institute of Bioproducts and Paper Technology, Graz University of Technology, Inffeldgasse 23 8010, Graz, Austria

[c] Univ. Grenoble Alpes, CNRS, CERMAV, 38000 Grenoble, France

[d] VTT Technical Research Centre of Finland Ltd., Biomaterial Processing and Products, Tietotie 4E, 02044 Espoo, Finland

[e] Graduate School of Environmental Studies, Tohoku University, 6-6-07 Aoba, Aramaki-aza, Aoba-ku, 980-8579 Sendai, Japan

[f] Division for the Establishment of Frontier Sciences of Organization for Advanced Studies, Tohoku University, 2-1-1 Katahira, Aoba-ku, 980-8577 Sendai, Japan

[*]Corresponding author. Tel: +358505124198. E-mail: michael.hummel@aalto.fi (Michael Hummel)

Number of pages: 14

Number of figures: 16

Number of tables: 2

Method of characterization of precursor fibers using WAXD and SAXS

WAXD data of the composite fiber were collected in the transmission mode setting of a CuKα X-ray instrument, SmartLab (RIGAKU) operated at 45 kV and 200 mA. The data of the composite fibers were collected in a powdered form, described previously in detail [1]. The diffraction data of cellulose, composite fibers, and keratin powder were calibrated for air scattering, sample holder, and inelastic scattering. Amorphous scattering contribution was subtracted through a robust smoothing procedure, and then the crystallinity of the material (*CI)* was estimated by the ratio of area of total (S_{total}) and amorphous (S_{bkg}) scattering:

$$CI = 100 \times \left(\frac{S_{total} - S_{bkg}}{S_{total}}\right) \tag{1}$$

The amorphous background corrected data of composite and keratin powder were used to subtract the scattering contribution of keratin in composite fibers. Since the scattering intensity of 2θ range around 8.9 (*Iker*) was dominantly generated by the diffraction of keratin crystal, we subtracted the keratin scattering profile from until the *Iker* of the composite fibers got close to 0.

After the subtraction of keratin contribution, crystal widths (CW_{hkl}) were obtained from the Scherrer equation (eq. 3) after a curve fitting procedure. The crystal widths were derived from the average from diffraction peaks of cellulose II: ($1\bar{1}0$), (110), and (020) [2].

$$CW_{hkl} = \frac{K\lambda}{\beta_{hkl}\cos\theta} \tag{2}$$

where S_{total} is the area of total intensity; S_{bkg} is the estimated area of background (from 9° to 50° 2θ); K is the shape factor (0.90); λ is the X-ray wavelength; and β_{hkl} is the full width of half maximum (*FWHM*) of the diffraction peak in radians and θ is the diffraction angle of the peak.

The azimuthal intensity profile was obtained from crystallographic (020) lattice plane (22.1° by 2θ), and was used to estimate the orientation distribution between fiber axis and crystallographic (020) lattice plane (ϕ_{020}):

$$\langle \cos^2 \phi_{020} \rangle = \frac{\int_0^{\pi/2} I(\phi_{020}) \sin \phi_{020} \cos^2 \phi_{020} \, d\phi}{\int_0^{\pi/2} I(\phi_{020}) \sin \phi_{020} \, d\phi} \tag{3}$$

Then Hermans orientation parameter (f_{WAXD}) was estimated between fibril axis and crystallographic c-axis:

$$f_{WAXD} = \frac{3\langle \cos^2 \varphi \rangle_{020} - 1}{2} \tag{4}$$

SAXS experiment were performed in a transmission mode of Xeuss 3.0 (Xenocs) CuKα X-ray instrument operated at 50 kV and 0.6 mA. Fiber samples were placed vertically on the sample holder and X-ray was radiated on the longitudinal side of the fiber. The sample chamber was kept under vacuum condition (= 0.16 mbar) during the measurement. The scattering intensity were recorded on a 2D detector Eiger2 R 1M (Detris) and the scattering intensity were corrected for cosmic background.

The azimuthal profiles were obtained in a scattering vector Q-range of 0.035 – 0.045 Å^{-1} with intensity corrected for the detector orientation, acquisition time and transmitted flux. The intensities were not corrected for the sample thickness. The obtained azimuthal profiles were fitted with a pseudo-Voigt function with a constant background. The fitted azimuthal profile was used to estimate the orientation distribution of the equatorial streak $\langle \cos^2 \varphi_{streak} \rangle$:

$$\langle \cos^2 \varphi_{streak} \rangle = \frac{\int_0^{\pi/2} I(\varphi_{streak}) \sin \varphi_{streak} \cos^2 \varphi_{streak}\, d\varphi}{\int_0^{\pi/2} I(\varphi_{streak}) \sin \varphi_{streak}\, d\varphi} \qquad (5)$$

Then it was converted into the orientation parameter (f_{SAXS}) in the same fashion as the Hermans orientation parameter is estimated from the equatorial diffraction assuming cylindrical symmetry:

$$\langle \cos^2 \varphi \rangle_c = 1 - 2\, \langle \cos^2 \varphi_{streak} \rangle \qquad (6)$$

$$f_{SAXS} = \frac{3\langle \cos^2 \varphi \rangle_c - 1}{2} \qquad (7)$$

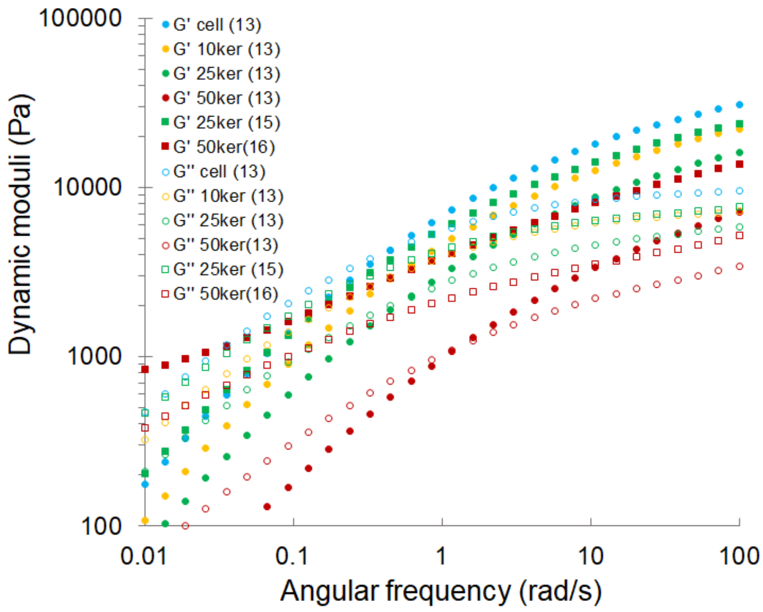

Figure S1. Dynamic moduli of the spinning dopes of cellulose pulp and mixture of cellulose pulp with 10, 25, and 50 wt% of chicken feather-derived keratin at 70 °C with a total polymer concentration of 13 and 15 wt.% in [DBNH]OAc. The dope with a total polymer concentration of 16 wt% does not exhibit a cross-over pointed within the measurement range.

Table S1. Contribution of keratin (%) from WAXD

Sample	Keratin contribution (%)
cell (13)	4.1 ± 0.3
10ker (13)	5.9 ± 1.4
25ker (13)	11.6 ± 0.8
50ker (13)	24.5 ± 6.6
25ker (15)	6.6 ± 2.3
50ker (16)	20.8 ± 6.0

Table S2. Mechanical properties of the keratin-cellulose composite fibers at DR 4 in the wet state

Sample	Tenacity (cN/tex)	Elongation (%)	Diameter, µm
cell (13)	35.0 ± 3.2	12.8 ± 1.5	17.6 ± 1.7
10ker (13)	32.9 ± 2.6	13.2 ± 1.5	16.5 ± 1.8
25ker (13)	33.6 ± 2.6	12.5 ± 1.6	16.4 ± 1.6
50ker (13)	23.2 ± 1.9	8.9 ± 1.0	15.2 ± 1.4
25ker (15)	32.1 ± 3.1	14.3 ± 2.18	17.1 ± 1.1
50ker (16)	22.2 ± 2.2	10.2 ± 1.05	17.1 ± 1.8

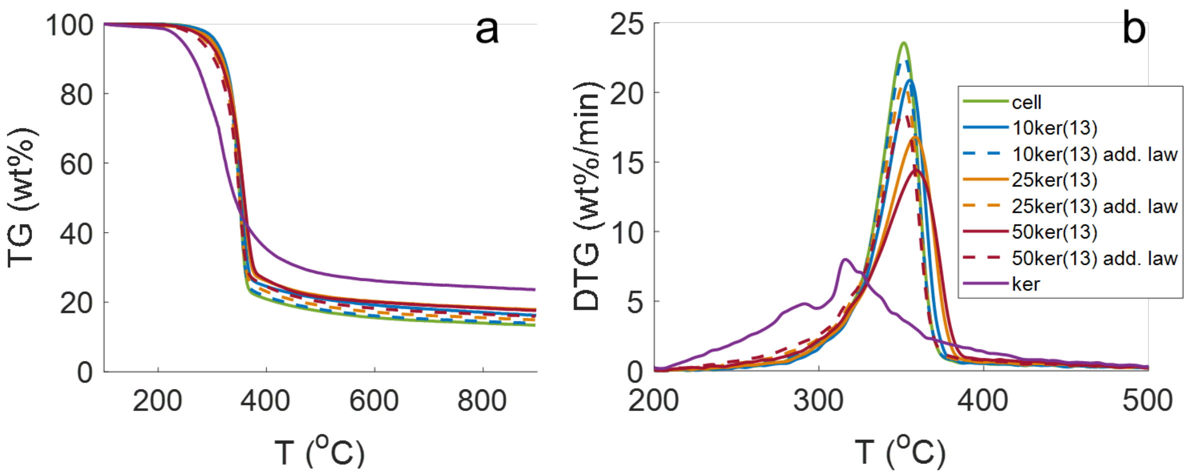

Figure S2. Comparison of experimental TG (a) and DTG (b) curves with the corresponding additive models of cellulose and keratin-containing fibers spun at DR 4.

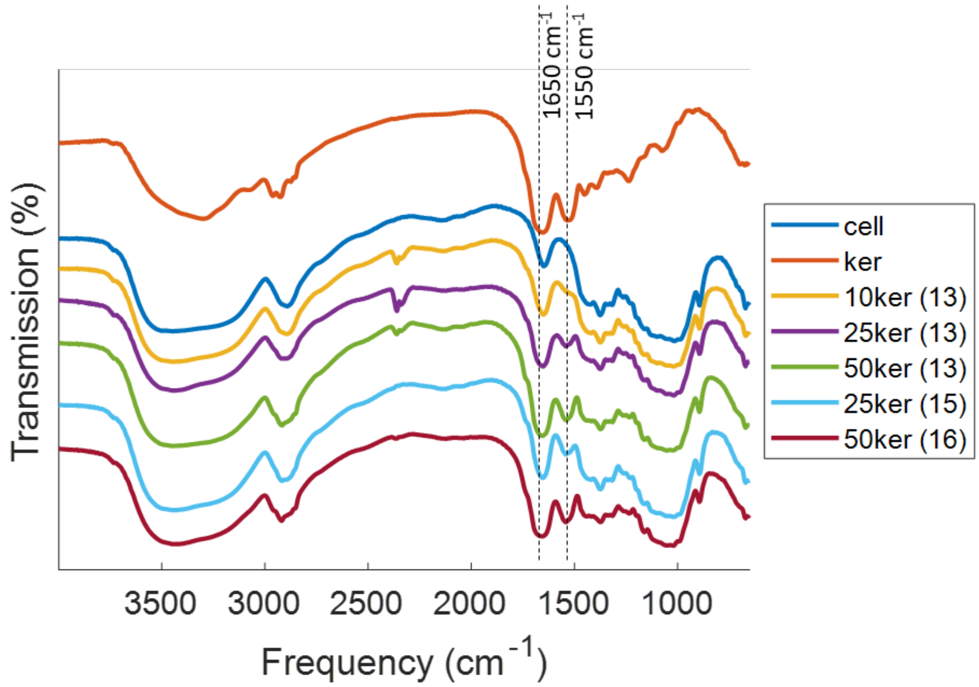

Figure S3. FTIR spectra of the precursor fibers with different total polymer concentration and initial concentration of keratin.

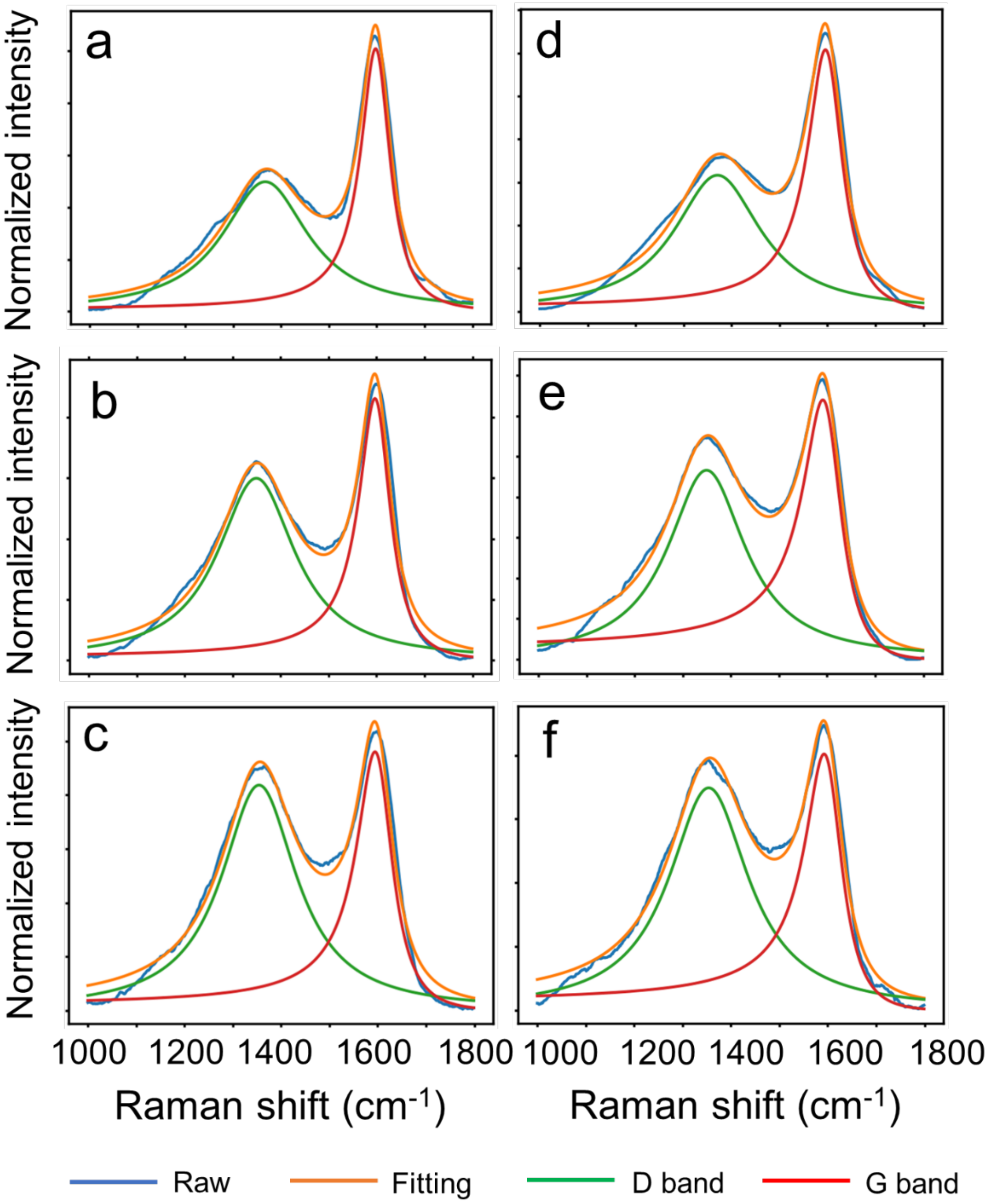

Figure S4. Raman fitting of carbon fibers produced from cellulose at: 500 °C (a), 700 °C (b), 900 °C (c); and produced from precursor fibers at 25 wt% initial keratin addition (25ker (15)) at: 500 °C (d), 700 °C (e), and 900 °C (f).

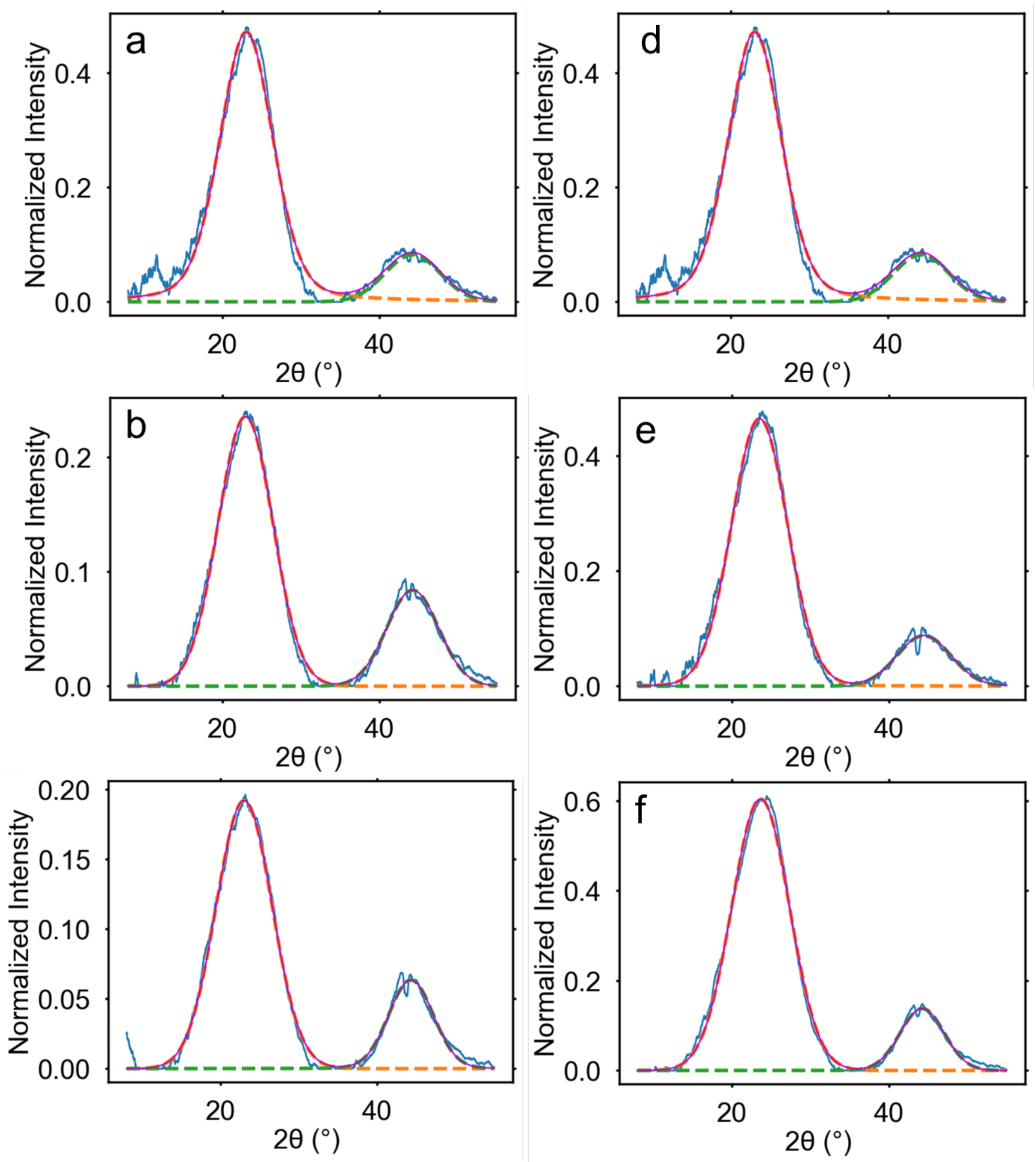

Figure S5. XRD fitting of carbon fibers produced from cellulose at: 500 °C (a), 700 °C (b), 900 °C (c); and produced from precursor fibers at 25 wt% initial keratin addition (25ker(15)) at: 500 °C (d), 700 °C (e), and 900 °C (f).

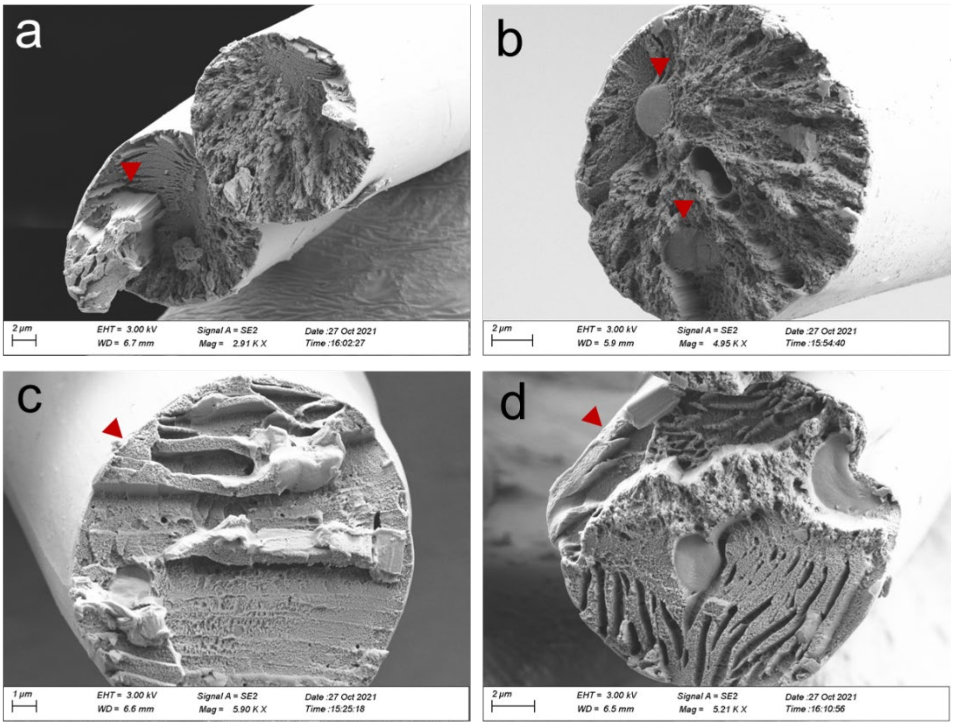

Figure S6. Cross-section SEM images from fractured fibers of 10ker (13) keratin composite fiber. The scalebar underneath the images represents 2 µm for a), b), and d), and 1 µm for c). Red arrowheads point toward the keratin domains, rotated arrowheads suggest the whole cross-section.

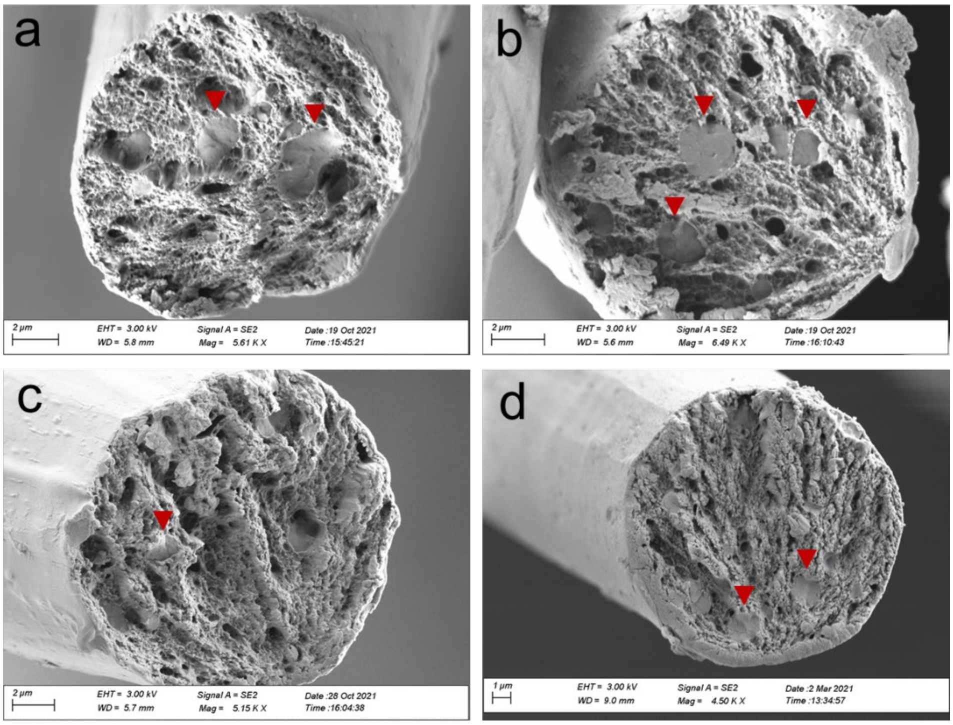

Figure S7. Cross-section SEM images from fractured fibers of 25ker (13) keratin composite fiber. The scalebar underneath the images represents 2 µm for a), b), and c), and 1 µm for d). Red arrowheads point toward the keratin domains.

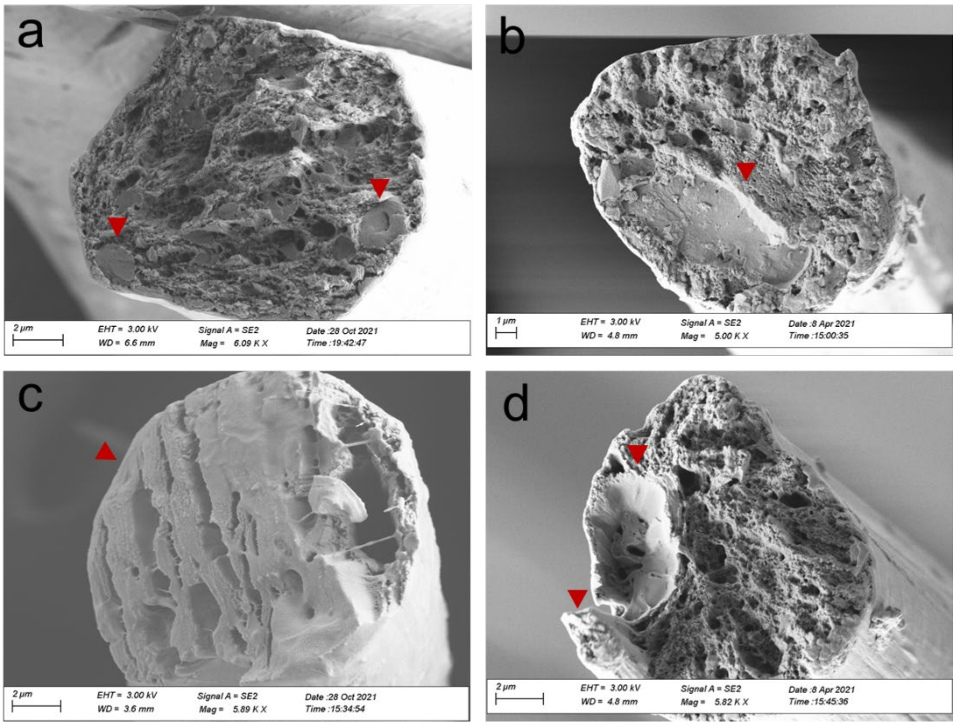

Figure S8. Cross-section SEM images from fractured fibers of 50ker (13) keratin composite fiber. The scalebar underneath the images represents 2 µm for a), c), and d), and 1 µm for b). Red arrowheads point toward the keratin domains, rotated arrowheads suggest the whole cross-section.

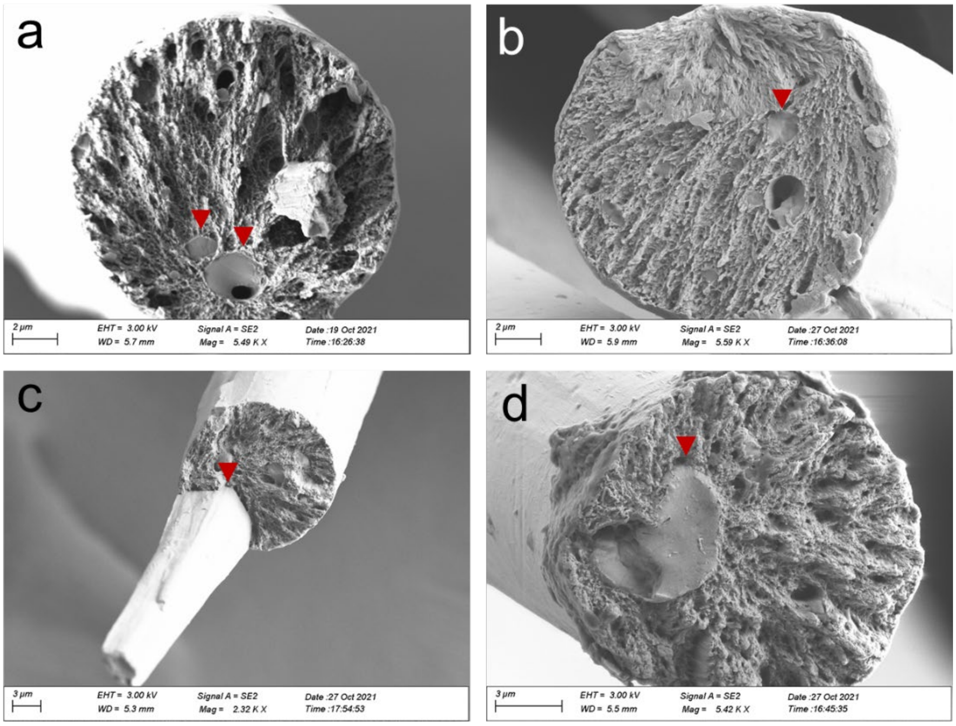

Figure S9. Cross-section SEM images from fractured fibers of 25ker (15) keratin composite fiber. The scalebar underneath the images represents 2 µm for a) and b), and 3 µm for c) and d). Red arrowheads point toward the keratin domains.

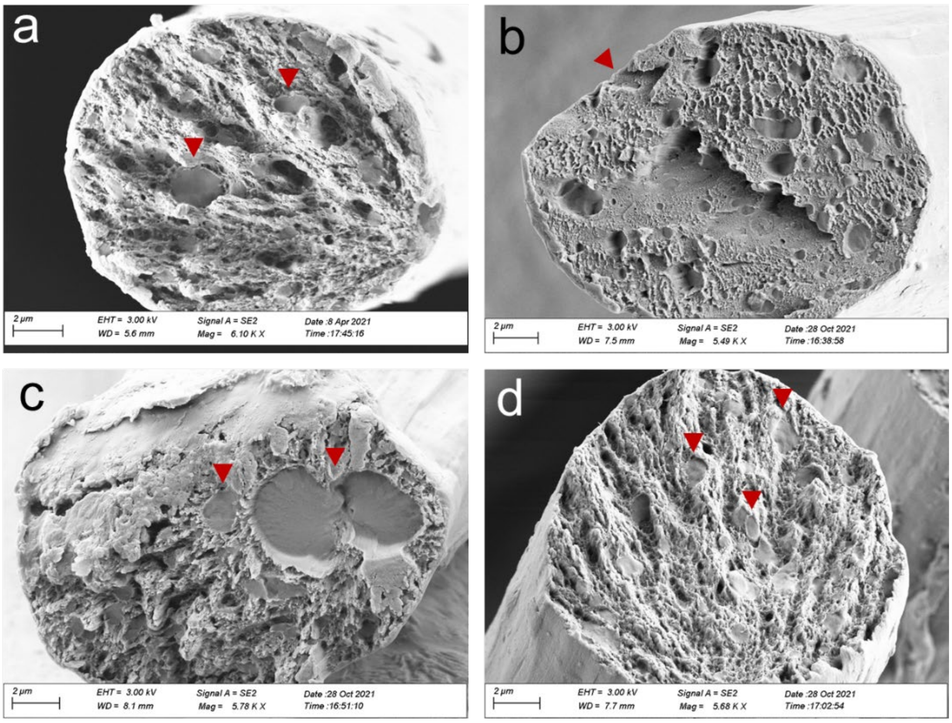

Figure S10. Cross-section SEM images from fractured fibers of 50ker (16) keratin composite fiber. The scalebar underneath the images represents 2 µm for a), b), c) and d). Red arrowheads point toward the keratin domains, rotated arrowheads suggest the whole cross-section.

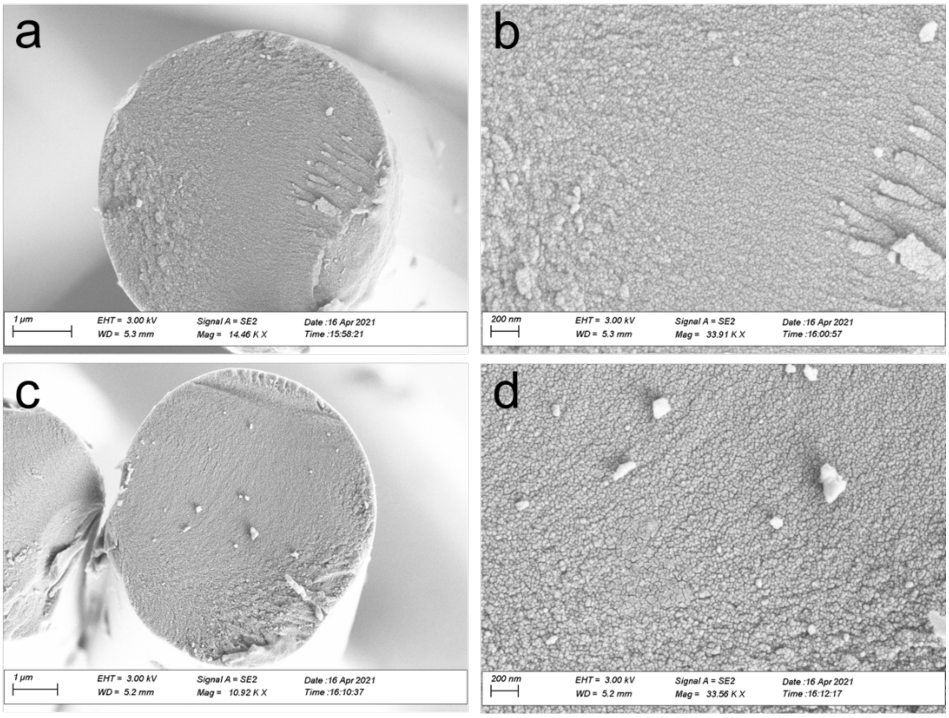

Figure S11. Cross-section SEM images of carbon fibers derived from cellulose fiber. The scalebar underneath the images represents 1 µm for a) and c) and 200 nm for b) and d).

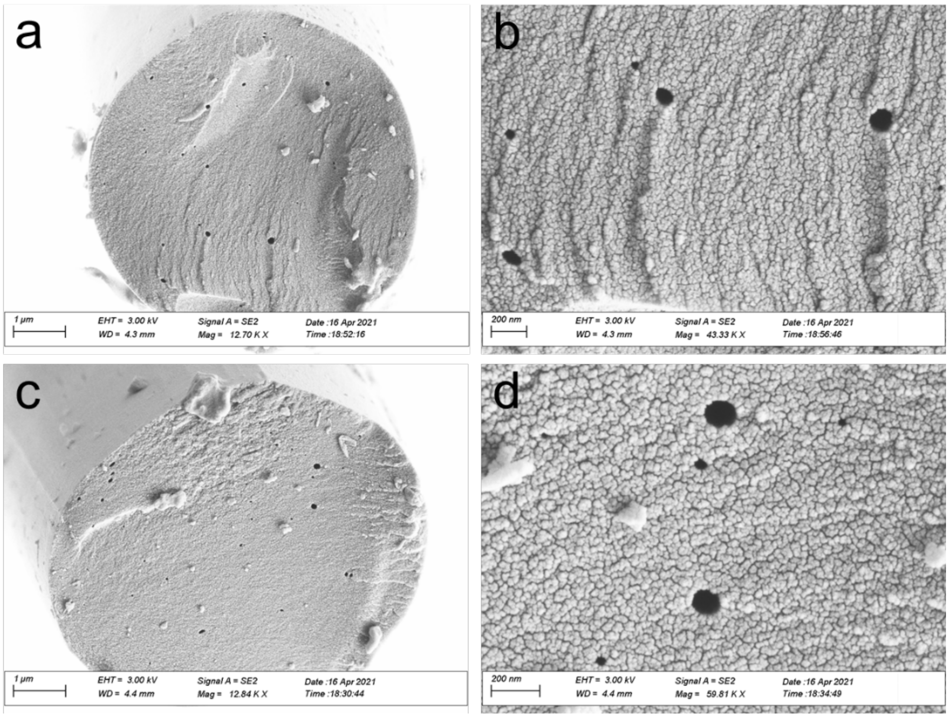

Figure S12. Cross-section SEM images of carbon fibers derived from 10ker (13) composite fiber. The scalebar underneath the images represents 1 µm for a) and c) and 200 nm for b) and d).

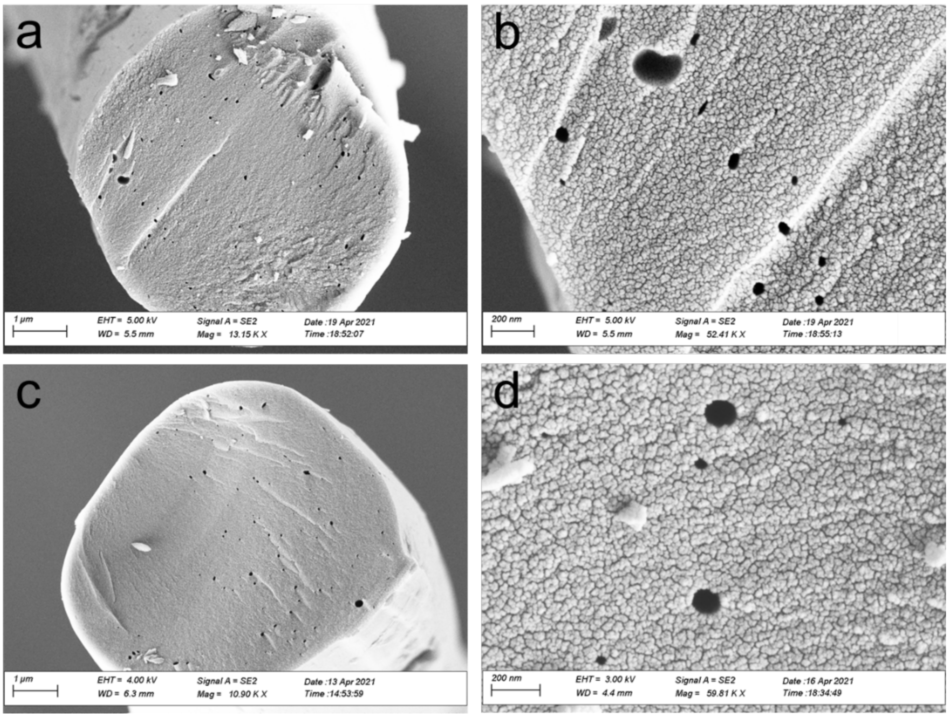

Figure S13. Cross-section SEM images of carbon fibers derived from 25ker (13) composite fiber. The scalebar underneath the images represents 1 µm for a) and c) and 200 nm for b) and d).

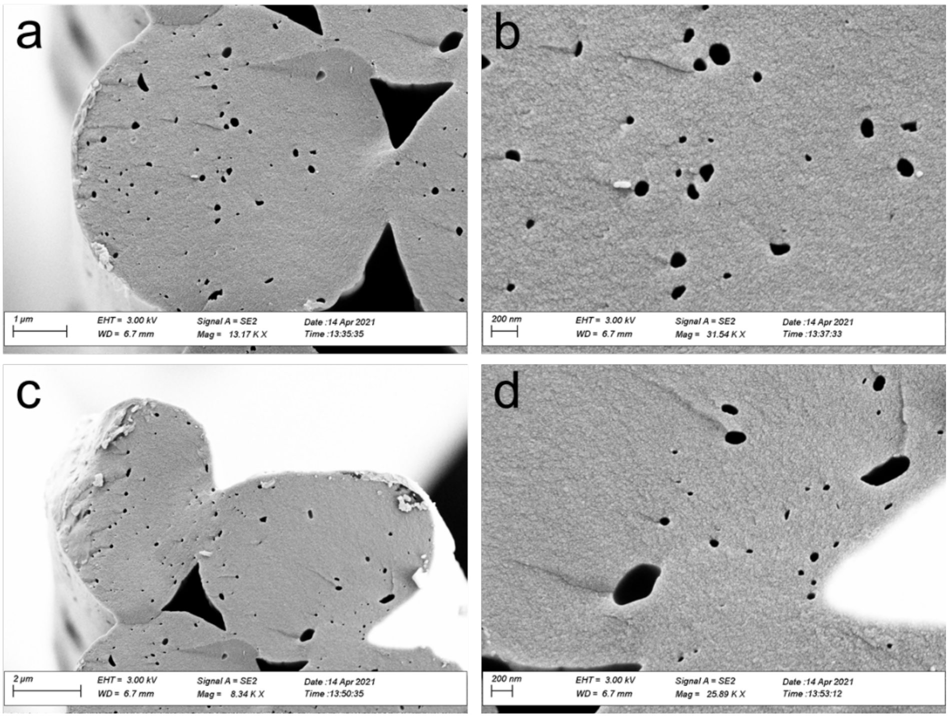

Figure S14. Cross-section SEM images of carbon fibers derived from 50ker (13) composite fiber. The scalebar underneath the images represents 1 μm for a) and c) and 200 nm for b) and d).

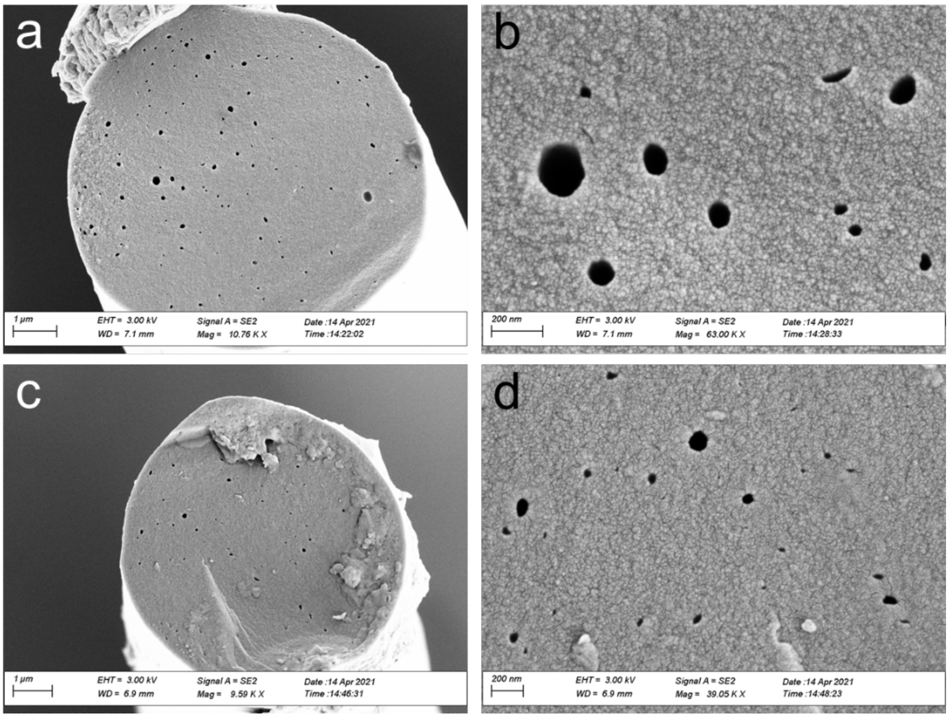

Figure S15. Cross-section SEM images of carbon fibers derived from 25ker (15) composite fiber. The scalebar underneath the images represents 1 μm for a) and c) and 200 nm for b) and d).

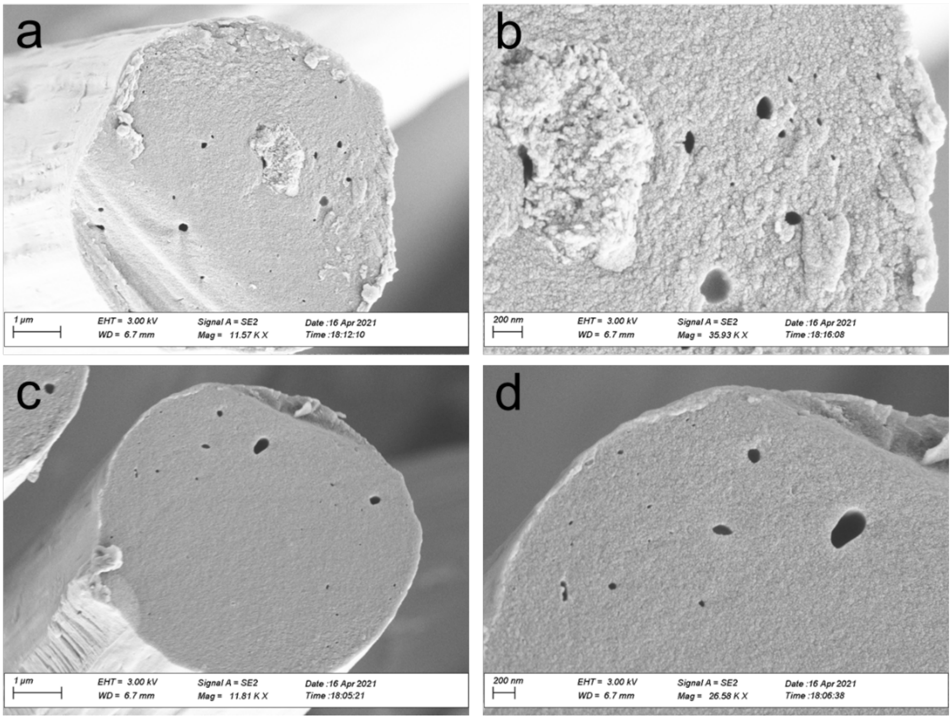

Figure S16. Cross-section SEM images of carbon fibers derived from 50ker (16) composite fiber. The scalebar underneath the images represents 1 µm for a) and c) and 200 nm for b) and d).

References:

1. Zahra, H., Sawada, D., Guizani, C., Ma, Y., Kumagai, S., Yoshioka, T., et al., *Close Packing of Cellulose and Chitosan in Regenerated Cellulose Fibers Improves Carbon Yield and Structural Properties of Respective Carbon Fibers.* Biomacromolecules, 2020. **21**: p. 4326–4335. DOI: 10.1021/acs.biomac.0c01117
2. Langan, P., Nishiyama, Y. and Chanzy, H., *X-ray structure of mercerized cellulose II at 1 Å resolution.* Biomacromolecules, 2001. **2**(2): p. 410-416. DOI: 10.1021/bm005612q
